# Proposal of a procedure to stratify the reidentification risk of medical data: RIMEDA

**DOI:** 10.1186/s12911-026-03475-4

**Published:** 2026-04-11

**Authors:** Sebastian Behre, Dorothea Kesztyüs, Sarah Schnabel, Tibor Kesztyüs

**Affiliations:** 1 Department of Medical Informatics, University Medical Center, Georg- August-University, Von-Siebold-Straße 3, 37075 Göttingen, Germany; 2 Department of Anaesthesiology & Surgical Intensive Care Medicine, Klinikum Werra-Meißner, Elsa-Brändström-Str. 1, 37269 Eschwege, Germany

**Keywords:** Routine medical data, Data sharing, privacy, Data security, Reidentification, Risk stratification, Pseudonymisation, Anonymisation

## Abstract

**Background:**

Routine medical data are highly valuable for secondary use, and data sharing is a prerequisite for pioneering research. Furthermore, since the advent of artificial intelligence and its application in various medical fields, such as decision-making and pharmacovigilance, the demand for real-world training data has steadily increased. However, the associated privacy risk, especially concerning reidentification, is extremely sensitive, and we are currently unaware of any standardised method to quantify it comprehensively.

**Methods:**

Assessing the reidentification risk of a data collection under examination requires the consideration and analysis of a complex system. To develop a holistic framework for stratifying this risk, an integrative approach is followed where the risk of deanonymisation is not considered mono-causally but includes various aspects. On the basis of a systematic literature review, factors and corresponding risks that are decisive in reidentification attacks are identified. These factors are grouped into overarching perspectives, and evaluation criteria are developed, facilitating the systematic grading of each risk factor by a data controller. Interactions between factors are visualised in entity‒relationship models (ERMs), and their direction and supposed magnitude are quantified in an influence matrix. Finally, on the basis of this matrix, a risk score and different indices are generated to evaluate the reidentification risk and facilitate possible countermeasures.

**Results:**

The reidentification risk comprises four general perspectives regarding data, knowledge, potential attackers, and technical/organisational aspects. The ERMs represent a complex system of clear interconnections between the factors of the respective perspectives. The final calculation is performed in the influence matrix based on the assessment of the data controller. The derivable indices and visualisations provide indications of particularly risk-driving components of a dataset and thus for targeted safety measures, such as generalisation, suppression and randomisation approaches. Experiments to determine the functionality of the method via published and verified reidentification attacks confirm the plausibility and selectivity of risk stratification.

**Conclusion:**

A quantitative assessment of the reidentification risk of a medical dataset, including the identification of risk drivers, is necessary and feasible. The proposed prototype must be further evaluated and will serve as the basis for the development of a software application.

**Supplementary Information:**

The online version contains supplementary material available at 10.1186/s12911-026-03475-4.

## Introduction

Medical data are being generated and collected in increasing quantity, speed, and variety in daily clinical practice and throughout the entire healthcare system. Through sophisticated techniques of modern data science, systematic analysis and processing, these data offer great potential for gaining insights for further research, general and individual healthcare, and public health. In particular, the rapid increase in the use of artificial intelligence (AI) in all areas of medicine is creating a growing demand for training datasets [1, 2]. However, there are data protection risks associated with the orchestrated processing and provision of health care data. In a much-cited paper, Sweeney (2002) demonstrated a linkage attack on a database with medical data from over 135,000 persons [3]. This dataset was considered “anonymous” and was made available to science and research for further use. Sweeney bought a freely available voter register, and by linking the zip code, date of birth and gender information from both data sources, she was able to locate the medical records of the state governor at that time. In the years following this report, the number of published attacks increased slowly until the mid-2000s but rose substantially in the following years, with 30 successful attacks published between 2011 and 2016 [4].

According to the Health Insurance Portability and Accountability Act (HIPAA) Journal website, in 2021, more data breaches than ever before were reported to the Office for Civil Rights (OCR), increasing further in 2022 [5]. In 2023, 725 data breaches were recorded, involving more than 133 million records that were disclosed or shared without authorisation. Patient-related medical data are considered very sensitive, and a lack of protection can, in addition to a loss of trust in the doctor–patient relationship, affect the willingness of patients to provide consent to further use their data or participate in studies. Furthermore, routine care data are considered “real-world data” and are becoming increasingly important for many different purposes [6]. Overall, digitally accumulating data volumes in healthcare systems result in great opportunities but also great responsibilities to protect these data and, in particular, the patients behind them [7].

To ensure data protection and research interests, medical data are pseudonymised or anonymised before release, i.e., potentially research-relevant information is removed or changed in such a way that the anonymised data meet data protection requirements but lose quality for research purposes [8]. “As little data protection as possible, as much data protection as necessary” or, as Gadotti et al. call this challenge, “The imperfect science of using data while preserving privacy” [9].

In this situation, it is very helpful for a data controller if the associated data protection risk can be estimated and stratified before releasing a dataset for secondary use. Ideally, particularly hazardous elements can be identified, and appropriate measures can be taken to counter this risk.

Reidentification risk is generally considered an underdeveloped area, although various methods for assessing reidentification risk for sharing individual-level data have been developed over time [10, 11]. In addition to the reidentification potential inherent in a dataset, the reidentification risk must also take into account how likely it is that the data will actually be misused to identify individuals [12]. In this context, Drechsler and Pauly refer to the Five Safes concept [13], according to which not only data-related measures such as anonymisation procedures should be examined, but also organisational and technical measures relating to projects, people, and settings. According to Morehouse et al., a risk assessment encompasses not only the potential for reidentification but also the associated consequences and should therefore go beyond a mere quantification of risk by using k-anonymity [14]. They point to the severity of consequences of disclosed data, which depends on the sensitivity of the content, e.g., information about mental health versus the results of a reaction time test. In this context, Sondeck and Laurent propose a comprehensive but “practical and ready-to-use methodology” to assess the severity and likelihood of reidentification at the attribute value level, inspired by the cybersecurity principle that primarily the most important risks should be addressed [15].

Other existing guidelines or tools for assessing the risk of reidentification of a dataset focus mainly on specific data and data types, e.g., free-text data [16], psychological data [17], data from clinical trials [18], or on characteristics of datasets, e.g., the extent of quasi-identifiers [19], and the quality of de-identification, i.e. anonymisation, efforts [20, 21], while external factors are hardly considered [22] or mainly as attack models with external data sources [10, 20, 23]. Furthermore, these tools should also be applied by appropriately trained users.

Meurers et al. focus exclusively on attackers, their motives, and the potential damage they cause, using a three-step process to establish a basis for assessing the risk of reidentification [24]. With the knowledge about these types of adversaries and the damage they can cause, they aim to support the evaluation of reidentification risks and contribute to the protection of the health data to be shared. Unlike others, Xia et al. calculate the probability of reidentification based on an attacker’s capabilities and an individual’s decision to disclose their participation in a dataset [10]. Accordingly, they show a significant reduction in risk with regard to voter registries and social media posts and conclude that the risk of reidentification is situation-dependent and that appropriate modelling could enable the sharing of biomedical data on a larger scale than currently possible.

In a more general approach, Ganiev et al., like Meurer, examine the reasons for reidentification attacks, attempt to understand the associated risk, assign it to corresponding scenarios, and refer to suitable anonymisation measures [25]. They also address the very important aspect of the data recipient as a “trusted recipient” who is bound, for example, by data use agreements. Morehouse et al. propose a decision tree with four dichotomous steps for determining appropriate data practices, as well as the use of open-source algorithms that apply intuitive anonymisation techniques to identify and mitigate privacy risks [14]. Simon et al. report on a toolkit for risk assessment and protection of data from health records based on careful review by experts. These data stewards review the data to be shared for linkability with external data sources, identify overlap patterns, and apply appropriate protection strategies and mechanisms for data sharing [23].

Taken together, these risk assessments are often conducted at the level of potential attackers and datasets, providing essential knowledge for protection against different types and points of attack on privacy. However, assessing the reidentification risk of a dataset under examination requires the consideration and analysis of a highly complex system. Therefore, this work follows an integrative approach where the risk of reidentification is not viewed as monocausal; rather, various aspects are included.

Three fundamental questions arise from the abovementioned demands for comprehensive data protection:

- Which factors are responsible for an increased data protection risk regarding the possible reidentification of one or more patients?
- Is there a way to determine the extent of the reidentification risk of a medical dataset and categorise or stratify it into risk classes?
- Can recommendations for risk reduction be derived that go beyond mere anonymisation or pseudonymisation?

On this basis, a comprehensive procedure is developed that enables an assessment of these questions and provides specific measures to improve the protection of sensitive data.

## Methods

For the development of the “ReIdentification risk of MEdical DAta” (RIMEDA) procedure, the literature was systematically searched to identify as many risk factors as possible that are associated with reidentification attacks on patient-related data. PubMed, IEEE Xplore and ACM Digital Library were searched from 2000 until November 13, 2024, for publications in English and German, with specific search words (reidentification, deanonymisation, attack, risk, attempt, health, personal, clinical, medical, data) using Boolean logic. To obtain comprehensive results, references in the included publications were also searched. The focus was on studies that examined data in tabular or relational formats, as medical data are predominantly organised in this way. The personal data examined in the studies should be pseudonymised or anonymised. Preference was given to publications of verified reidentification attacks to enable a subsequent assessment of the risk potential of the attack vectors used in the sense of a proof of concept. Reidentification attacks targeting image or signal data cannot be considered in this basic form of the RIMEDA approach because of increased complexity. However, the information considered was type, scope, origin of the attacked data, context of its publication, methods and associated factors that led to deanonymisation, and reflections and factors utilised to assess the risk. The literature contains multiple studies on the reidentification risk of medical datasets from several aspects, but no holistic approach could be found.

### Methods for risk stratification

#### Entity relationship models

Entity relationship models (ERMs) are mainly used in the field of database programming, where they describe in a structured and formal way a certain “section of the real world” in the context of a semantic or conceptual data model.

**Fig. 1:**
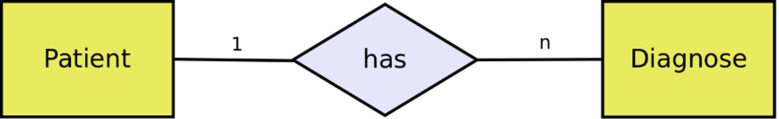
Entity relationship model with a 1:n relationship without attributes

Attributes are used to describe an entity or a relationship in concrete terms. Patients, e.g. have a date of birth, a gender, an address, etc.; tumours are linked to International Classification of Disease (ICD) codes, laboratory parameters and drugs. The specific expression of an attribute is called an attribute value; the possible range of values is called a domain. Figure 1 shows a simple ERM.

#### Modelling of influencing factors

On the basis of the systematic literature review, factors and corresponding risks that are decisive for reidentification attacks are identified and generalised into overarching entities, with attributes that concretely describe the entity. In ERMs, generalised entities are known as supertypes, but in the following, they are referred to as perspectives.

A numerical value is assigned to each attribute to represent the respective strength of influence on the “reidentification risk” system. A four-digit scale with absolute values between “0”, which represents no influence, and “3”, which represents strong influence, is utilised with criteria for assigning scores as objectively as possible. In the next step, the interactions between the factors are then visualised in ERMs to represent the relationships on a qualitative level and thus show the “nature” of the respective relationship [26]. The interactions in the ERMs are subsequently assigned numerical values in an influence matrix according to their strength and direction [27]. This results in indices both for each individual entity and across components, which can be used to describe the dynamics of the reidentification risk. To demonstrate the reidentification risk of a specific dataset in concrete terms, the assigned “weights” of the entities are combined with the “dynamics” of the system. The more pronounced a factor is, the stronger its effect on the system “reidentification risk”. Finally, the quantitative recording, presentation and evaluation of the reidentification risk of a medical dataset is offered in corresponding scores and graphical representations. The prototype for this risk stratification model is realised in a common spreadsheet program, which can be found in the additional files (behre_rimeda_prototype.xls).

##### Conceptualisation of an influence matrix

This specific method follows an approach for capturing and representing complex systems within the framework of so-called “networked thinking” within systems theory [27]. In a two-dimensional matrix, the previously determined influencing factors are entered in the same order in the header column or row and thus compared. On the basis of the ERMs, the interactions identified and their respective extents between the individual pairs of influencing factors are examined and categorised. The estimated strength of the impacts is represented by assigning numerical values in the interval [-3; 3]. The moduli of the numerical values describe “0” = missing, “1” = weak, “2” = medium, and “3” = strong impact of one factor on another. A positive sign represents a parallel, whereas a negative sign represents the opposite development of the factors.

The so-called active and passive sums are subsequently determined. The active sum, or cumulative influence strength of a factor on the system, is the sum of the absolute values in the corresponding row of the influence matrix. Accordingly, the passive sum equals the sum of the absolute values in the corresponding column of the matrix.

The Q-value, as the product of the active and passive sums of an influencing factor, illustrates how strongly an entity is cross-linked with the other system components and thus has an effect on the system. A positive Q-value indicates a risk-increasing effect, and a negative Q-value indicates a risk-reducing effect. The higher the Q-value is, the more suitable the factor under consideration (assuming controllability) is as a “lever” for influencing the system [28].

The total sum of the passive and active sums serves as a measure of the overall “reidentification risk” system. The stronger the influencing factors and thus the probability of occurrence of said risk are developed as a whole, the larger this sum, i.e., the risk score becomes. The influencing factors can be divided into four categories according to the ratios of their active and passive sums [27].

1. Active/impulsive influencing factors: characterised by pronounced active sums and rather low passive sums, they influence the system more strongly than they themselves are influenced by it.
2. Reactive/passive influencing factors: characterised by strong influenceability or high passive sums and rather low active sums, they are influenced by the other entities to a greater extent than influencing the system itself.
3. Critical/dynamic influencing factors: exert a significant influence and can be strongly influenced due to a pronounced interconnectedness with the other factors of the system.
4. Inertial/buffering influencing factors: represented by overall low active and passive sums and, owing to their low level of interconnectedness, have hardly any significant active or passive influence on the system and can therefore be regarded as isolated.

More detailed information is provided as an additional file (behre_rimeda_supplementary_information.pdf).

## Results

### Modelling of risk factors

Owing to the very small number of described reidentification attempts involving patient data, the literature analysis was expanded beyond purely medical data. According to the results of the literature review, the influencing factors (entities) were assigned to four overarching perspectives: the data, knowledge, attacker, and technical/organisational perspectives.

#### The data perspective

This perspective first includes all those entities that can be derived from the data via mathematical methods and thus represent precise metrics. In addition, quasi-identifier combinations considered to be particularly risky are grouped together to form the entity “vulnerable quasi-identifier”.

Table 1 provides an overview of the entities from the data perspective.

**Table 1:** Data perspective (supertype)

Entity	Attribute	Domain	Weighting
Uniqueness	Proportion of total number of records	[0%, 100%]	0–3
Similarity	Number of similar records	[0%, 100%]	0–3
Vulnerable Quasi-Identifier	Length of stay	[not] included in the dataset	0–3
	Treating physician		
	Diagnosis		
	Date of admission		
	Date of birth		
	Sex		
	Zip code		

#### The knowledge perspective

This perspective refers to the level of additional knowledge required to restore a personal reference. The contained entities cannot be represented by a specific function but are subject to a certain fuzziness with respect to their respective attributes and domains.

Table 2 shows an overview of the entities from the knowledge perspective.

**Table 2:** Knowledge perspective (supertype)

Entity	Attribute	Domain	Weighting
External data sources	Type	undetermined	1–3
	Suitability	[unsuitable; suitable]	
	Availability	[low; medium; high]	
Background/contextual information	Type	undetermined	1–3
	Suitability	[unsuitable; suitable]	
	Availability	[low; medium; high]	
Metadata	Type	see “metadata”	0–3
	Suitability	[unsuitable; suitable]	
	Availability	[low; medium; high]	
Data inference	Potential	[low; medium; high]	1–3

#### The attacker perspective

This perspective assesses the influence of a potential attacker on the reidentification risk.

Table 3 provides an overview of the entities from the attacker’s perspective.

**Table 3:** Attacker perspective (supertype)

Entity	Attribute	Domain	Weighting
Motivation	Degree	[low; medium; high]	1–3
Skills	Degree	[low; medium; high]	1–3
Resources	Degree	[low; medium; high]	1–3

#### The technical/organisational perspective

Finally, environmental topics are unified from a technical/organisational perspective. Only a few mentions of this topic were found in the literature, but statistics provided by the HIPAA Journal on data breaches, among others, show that this perspective should not be neglected [5, 29].

Table 4 provides an overview of the entities from the technical/organisational perspective.

**Table 4:** Technical/organisational perspective (supertype)

Entity	Attribute	Domain	Weighting
Awareness	Degree	[low; medium; high]	-3; 1–3
	Degree of Implementation	[low; medium; high]	
Methods	Type	undetermined	-3; 1–3
	Quality	[low; medium; high]	
Data security	Degree of Implementation	[low; medium; high]	-3; 1–3

### Entity relationship models of the risk factors

In the ERMs, correlations and dependencies between the factors influencing the reidentification risk of patient data are presented. Each perspective is examined for its interactions with the other perspectives. Based on these models, an influence matrix is built and then used to stratify the reidentification risk.

#### Interrelationships of the entity “uniqueness” of the data perspective

The uniqueness and similarity of data records are closely linked properties. There is a strong causal relationship between the uniqueness characteristic and the entity of “vulnerable quasi-identifiers”. These identifiers are classified as particularly risk-driving since they are associated with a pronounced uniqueness related to the entire dataset.

Unique datasets are a prerequisite for linkage attacks on the “external data sources”, “background knowledge/context”, and “metadata” entities of the knowledge perspective. The more unique the dataset is in this context, the more accurate the results a corresponding attack can deliver; thus, a strong interaction can be assumed. There is also a corresponding interaction with respect to possible data inference, but this depends on the data under consideration and must therefore be regarded as somewhat weaker. For example, an associated diagnosis can often be inferred from a unique combination of medication and procedures.

From an attacker perspective, it can be assumed that knowledge of the possible existence of unique datasets can greatly increase the motivation to attempt a reidentification attack. Moreover, it drives the development and improvement of an attacker’s skills. Finally, identifying unique data consumes attacker resources.

If the risks of unique datasets are known to the responsible persons of an organisation, this may have an increasing effect on their awareness. Analogous to the attacker perspective, knowledge of this entity acts as a driver for the development and improvement of protection approaches. Areas of data security are also affected. Accordingly, special measures related to the aspect of “confidentiality” can be initiated, for example.

**Fig. 2:**
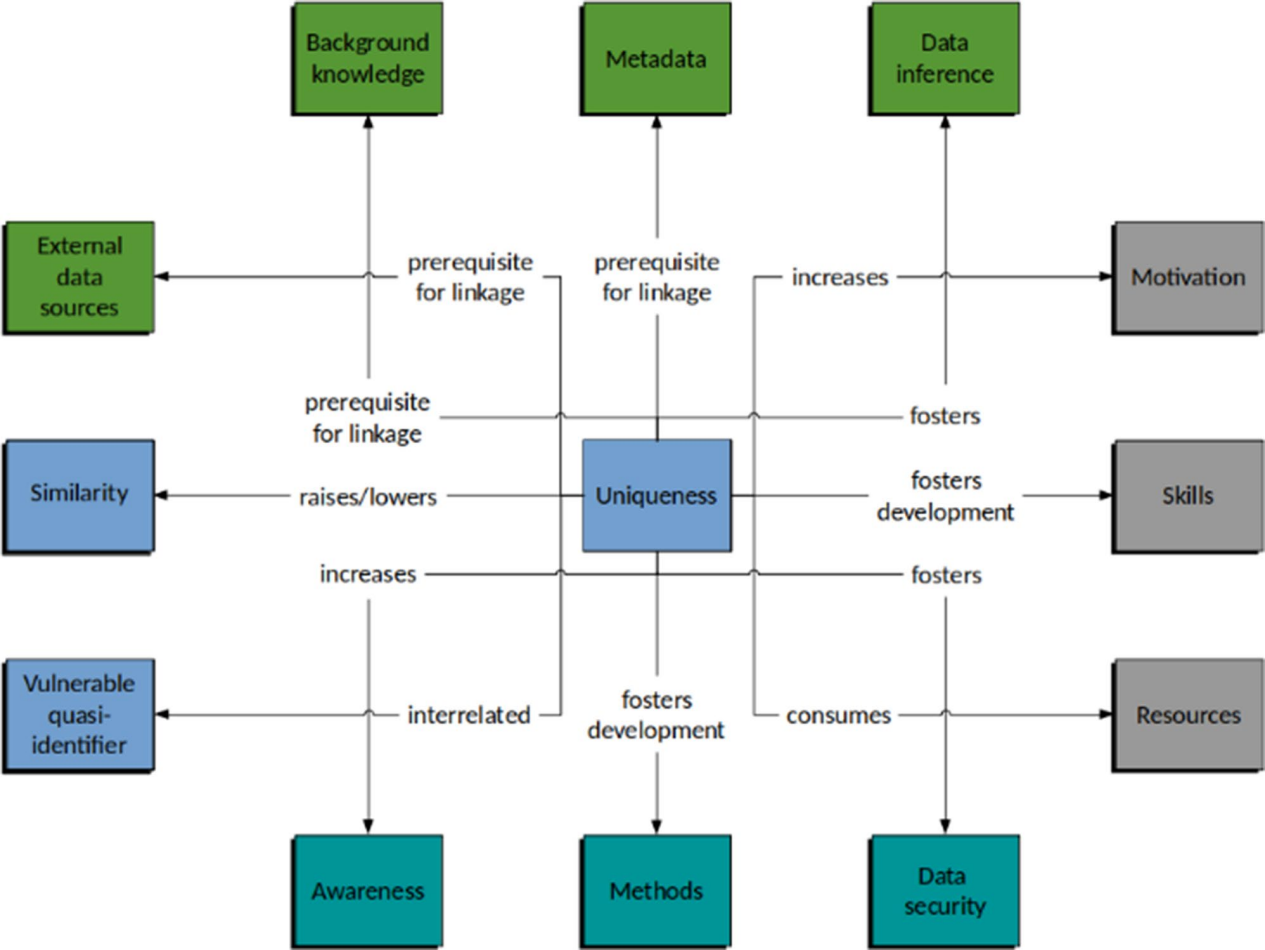
Interrelationships with other entities and their qualitative nature in the case of the entity “uniqueness”

The relationships described here are depicted in the ERM in Fig. 2.

The descriptions of the interrelationships of all other entities with the respective graphical representations is provided in an additional file (behre_rimeda_supplementary_information.pdf).

## Influence matrix

The interrelationships between the entities of the respective perspectives clearly show cross-linking, which results in a picture of a complex system. However, ERMs can only describe existing interactions between entities qualitatively, not quantitatively. Hence, to systematically show the dynamics of these connections, the so-called influence matrix is created, as described in the Methods section.

To present a risk assessment of a concrete dataset, it is necessary to combine the influence matrix with the weighting components of the individual entities. For this purpose, the absolute values of the interactions in the matrix are multiplied by the assigned weighting scores of the entities. The more pronounced a factor is, the stronger its effect on the “reidentification risk” system, e.g., an attacker with high methodological skills has a stronger effect on the system than does an attacker with low methodological skills. The resulting indices are then plotted in evaluation diagrams (see below).

**Fig. 3:**
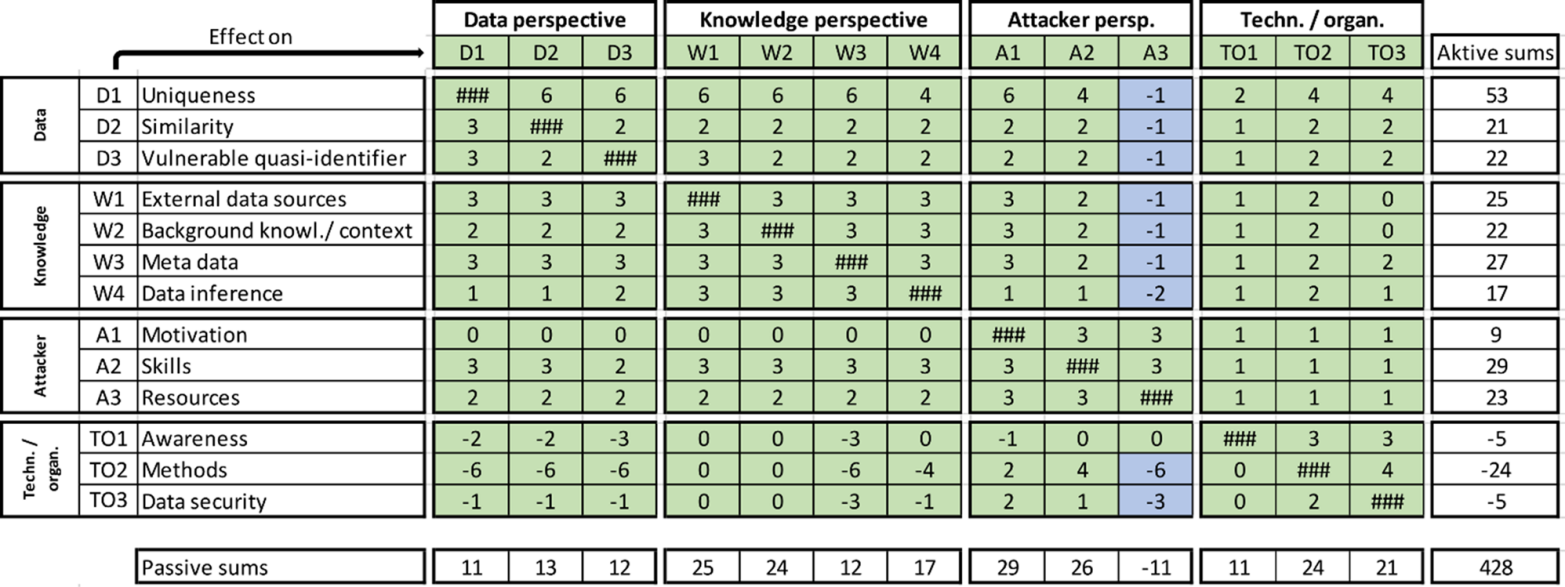
Matrix of influence for calculation of the reidentification risk. The reidentification risk is seen as a complex system of interacting factors. These mutual interactions are presented here in quantitative terms. The modulus of the respective value determines the strength of the effect of one factor on another, whereas the sign indicates the direction of the effect, i.e., whether one factor strengthens another or reduces the influence of another component on the system. Entities D1 and TO2 impact the system with double point values

On the basis of the ERMs and related explanations, the influence matrix depicted in Fig. 3 is proposed for the “reidentification risk of patient data” system. The numbers in the cells correspond to the weighting of the correlations identified in the respective ERMs.

The following applies: 0 points correspond to no effect; 1 point corresponds to a weak effect; 2 points correspond to a medium–strong effect; and 3 points represent a strong effect. The numbers represent weighting components.

### Risk assessment

Two steps are required to assess the reidentification risk of a specific dataset:

1. Before calculating the reidentification risk of a specific dataset in the influence matrix, the data controller must provide the respective weighting scores of the entities from the risk perspective.
2. Once the scores have been entered, the absolute values determined are multiplied by the absolute values of the effect relationships. The indices of the influence matrix change and show the magnitude of the reidentification risk.

The total assessable risk of unwanted reidentification falls between 28 and 1670 points, with a risk range of 1642 points.

**Fig. 4:**
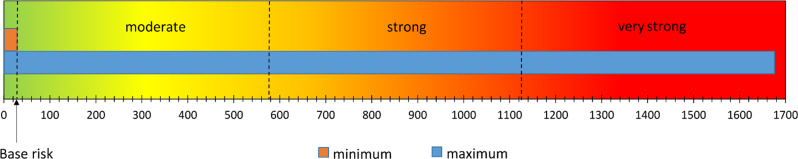
Graphical representation of the reidentification risk score, which illustrates the overall degree of severity. Here, the minimum (basic) and maximum possible values are shown on the basis of the total sums of the active and passive sums

Figure 4 illustrates the maximum and minimum representable total risk scores

**Fig. 5:**
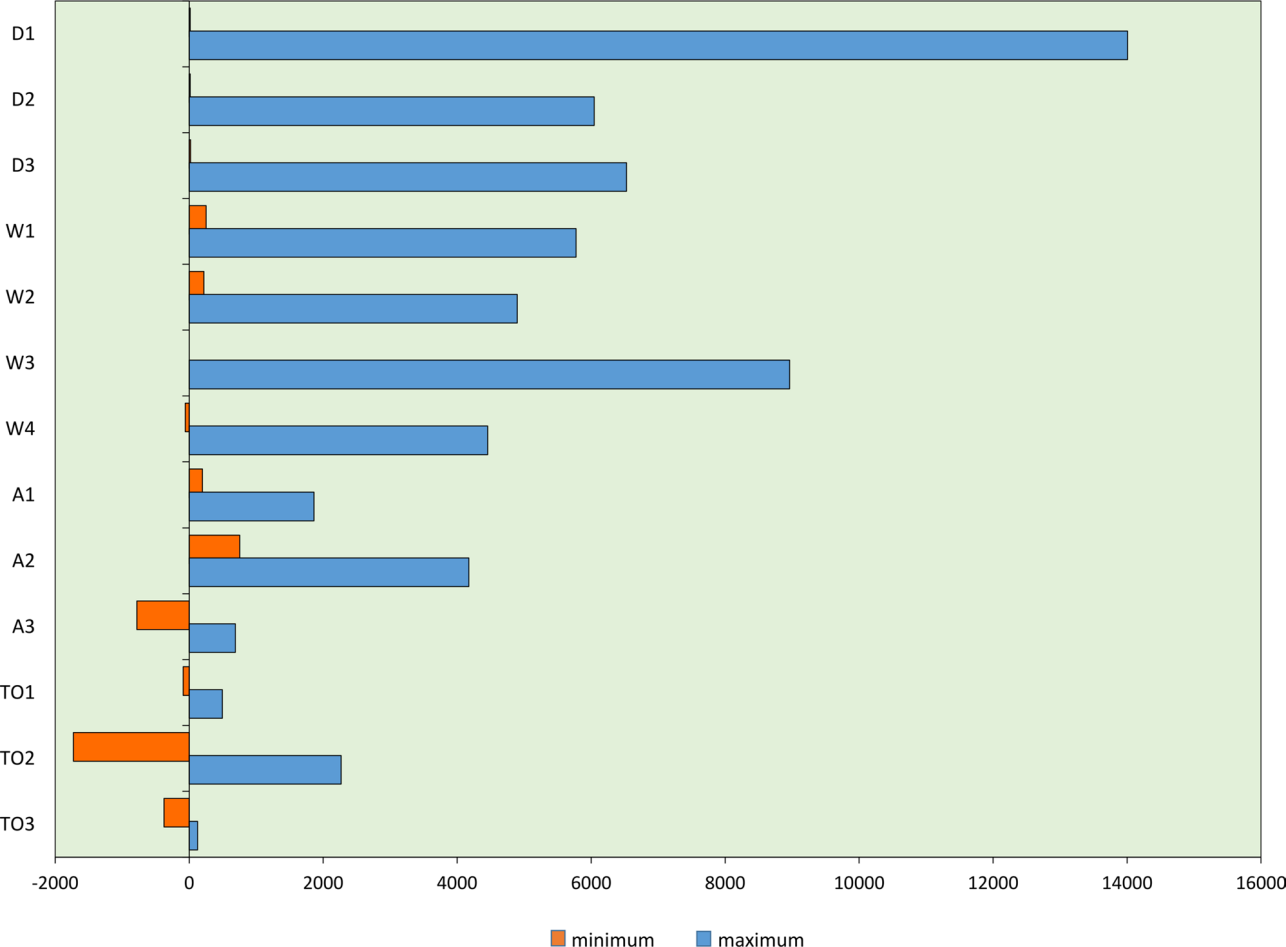
Graphical representation of the maximum and minimum Q-values of the influencing factors. The Q values of the individual entities are shown. The more pronounced this value is, the stronger the cross-linking of the factor under consideration and the greater its influence on the system. This factor (or factors) therefore provides an indication of the adjustments that need to be made to control the risk of reidentification (provided that the factor is controllable)

The Q-value assessment chart provides useful indications of how the reidentification risk of a specific dataset can be effectively reduced. Figure 5 shows that, at maximum discernible risk, dataset uniqueness (D1) and metadata (W3) are very strong risk factors and should be addressed accordingly. In contrast, at minimum risk, the method quality (TO2) has a significant inhibiting effect on the system and is therefore the most likely cause of the low risk level.

The active sums of the influencing factors range from − 72 to + 161 points, and the passive sums range from − 34 to + 108 points. The active and passive sums of a factor are rounded up for better presentability and plotted as a pair of values in a Cartesian coordinate system: the active sum on the abscissa and the passive sum on the ordinate. The diagram has subdivisions of equal size, which represent the increasingly stronger expressions of the value pairs (and thus also of the total risk). This representation makes it possible to analyse the position of a specific factor in the system and to assess the extent to which it actively influences the risk or how strongly it is influenced by other components. Hence, the presentation complements the visualisation and usefulness of the Q-values.

**Fig. 6:**
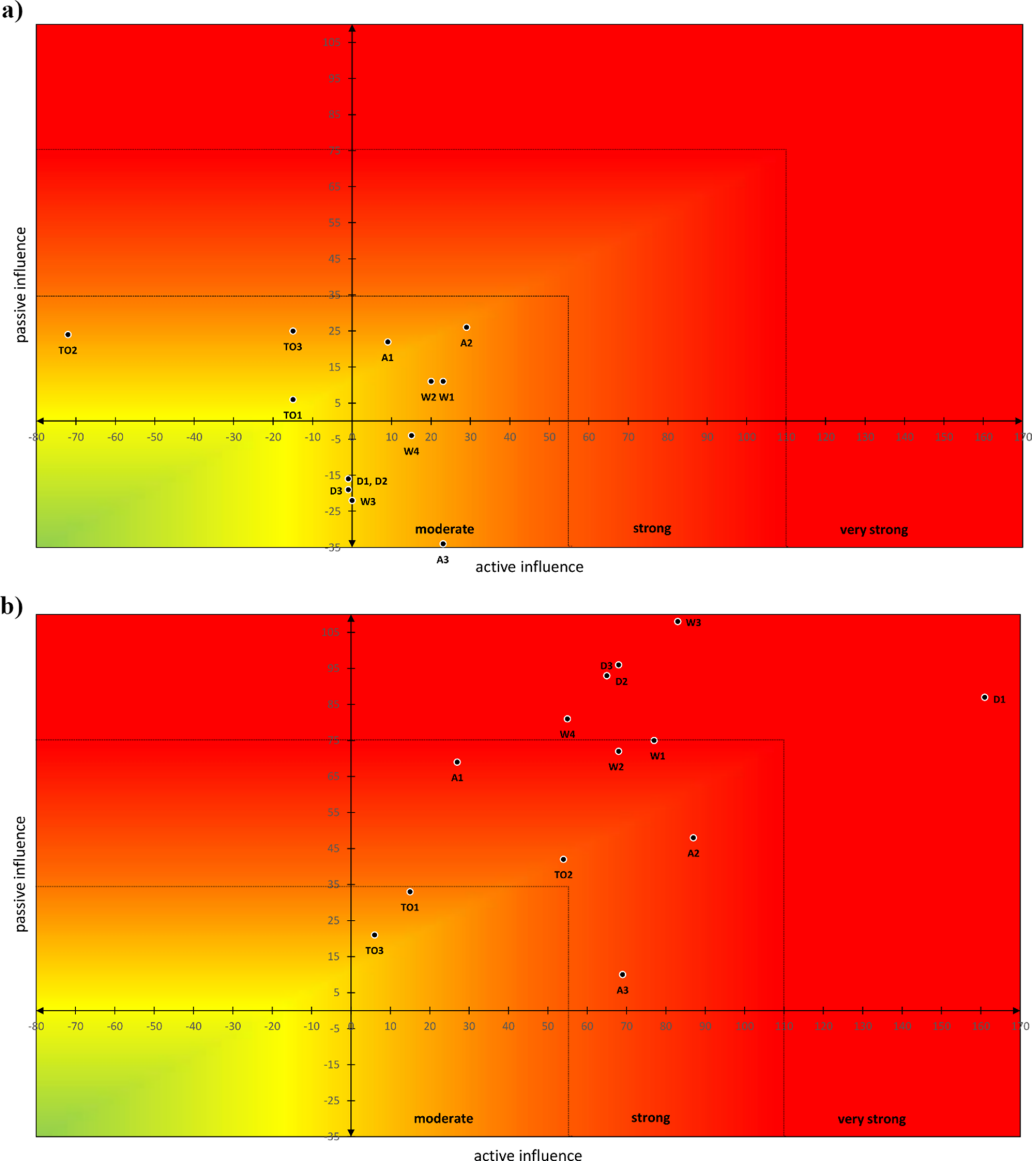
**a**. Differentiation of the smallest reidentification risk according to influencing factors. **b**. Differentiation of the highest reidentification risk according to influencing factors

Figures. 6a and 6b illustrate the smallest and the highest possible risk

These figures demonstrate the resulting reidentification risk after the weighted factors have been applied to the system model, with the minimum (baseline) values shown in Fig. 6a and the maximum values in Fig. 6b.

The x-axis illustrates the active influence of the elements, with positive values indicating an increase in risk and negative values indicating a reduction.

The ordinate shows the extent to which a factor is influenced by the other entities. Positive values indicate that the effect of a factor is amplified by other factors. Negative values indicate that the effect of the aspect under consideration is weakened by the other system components.

The more elements there are in areas with high active and passive sums, the more pronounced the resulting risk.

Taken together, these results demonstrate the application of the system-theoretical method in the influence matrix as an instrument for risk stratification. On the basis of the concrete influence matrix, derivable indices and specific visualisations of the deanonymisation risk of a dataset are presented.

The proposed use of the RIMEDA procedure, guidance and explanation for assigning a score to an attribute to indicate the respective strength of the influence on the “reidentification risk” system; complete information on all ERMs; further explanation of the influence matrix; and recommendations for appropriate countermeasures can be found in the additional file (behre_rimeda_supplementary_information.pdf). Furthermore, an evaluation of the procedure based on predefined scientific quality criteria is provided by means of retrospective application tests of two published successful reidentification attacks.

## Discussion

In this work, the reidentification risk of a medical dataset is considered a multifaceted challenge to the protection of sensitive data and the individual privacy of the data donors. The view taken was therefore as comprehensive as possible, leading to an integrative approach with a multicausal risk consideration. Influencing factors and corresponding risks were identified in a thorough literature review, grouped thematically into hierarchical organised perspectives, and defined with specific, assessable attributes. We evaluated and visualised the interrelationships between risk factors from different perspectives in ERMs, summarised and quantified them in an influence matrix, and finally visualised and created a framework to make the complex system “reidentification risk” comprehensible. On this basis, we developed the RIMEDA procedure for quantifying and stratifying the reidentification risk of a medical dataset in a fundamental version that can be implemented by a well-trained data controller.

The identified risk factors were generalised in overarching perspectives: the data, knowledge, attacker, and technical/organisational perspectives. With regard to the latter, although an organisational guideline works differently than a technical barrier, both are closely linked within an institution. Hence, technical and organisational aspects were grouped together as topics that, compared to the other perspectives, are less directly related to attackers or attacks, but rather represent related, overlapping, and interconnected environmental issues. The associated risk factors or entities listed are clearly related to organisational and technical aspects. Furthermore, these two aspects are crucial for risk reduction, which is reflected in the influence matrix by point deductions or negative weighting. However, environmental aspects are rarely addressed in common assessments of reidentification risk.

Although there has been some scientific discourse with regard to risk assessment, most concepts are generally associated with limitations, and the area of reidentification risk is still considered underdeveloped [11]. The available methods are, in principle, based on reidentification attacks and consider only a few aspects of the risk or view it from hardly more than the perspective of the attacker or the data controller [10]. Various approaches generally consider only partial aspects of the deanonymisation risk, and corresponding risk assessments are often carried out in a predefined context. For example, general models are proposed for risk assessment in which the context of a specific situation is included in the assessment [30]. Furthermore, the uniqueness or distinctiveness of datasets is regarded in many studies as the main criterion for classifying the reidentification risk [10]. Possible reasons for this could be the objectivity and the low effort required to calculate this characteristic, e.g., the problem of easily distinguishable medical laboratory data [31]. The characteristic of uniqueness is usually supplemented by a number of other aspects, which together form a “risk indicator”. The same applies for quasi-identifiers, which are particularly suitable for reidentification because of their pronounced uniqueness and great potential for data inference [32].

Less objectifiable aspects, such as the sociopsychological component of reidentification risks, which focus on and characterise attackers, are also used for risk assessment [33]. From a technical perspective, however, many publications only indirectly address the risk of reidentification, for example, by examining the cryptographic hardness of algorithms or other aspects of IT security [34]. Weaknesses in these methods are then associated with increased data protection risk.

These explanations show that risk assessment approaches are generally developed for specific datasets that are also considered under defined environmental parameters. This makes it possible to make a relatively concrete but also very specific or monocausal classification of deanonymisation risk of patient data. Outside the defined parameters, however, the proposed methods are presumably no longer meaningful to the original extent. The restriction or focus on objectifiable or calculable characteristics neglects other relevant influencing factors with regard to a possible reconstruction of personal references.

Hence, to the best of our knowledge, RIMEDA is the first method to offer an integrative approach for modelling and stratifying this special data protection risk without restrictive situational and data-related prerequisites. The reidentification risk is understood as a complex system based, among other factors, on components that are usually considered only individually. Using modelling techniques and the system-theoretical instrument of the influence matrix, these factors are inserted into a holistic, logical context to derive a statement about the probability of deanonymisation. In addition, the indices derived from the influence matrix enable a closer look at individual risk components.

### Limitations of the RIMEDA procedure

The probability of the deanonymisation of medical data is highly influenced by factors that are difficult to record and quantify objectively. This conflicts with the need to specify and assess the risk-driving elements as specifically as possible to obtain a reliable risk prognosis. The operationalisation of the entities “awareness”, “external data sources”, “background information”, and “data inference” represents a challenge in stratifying the reidentification risk of medical data. When modelling these characteristics, the most objective and comprehensible criteria possible were provided for assessment, but nevertheless, only an estimate of the respective characteristics is possible.

Although the influence matrix used, including all effect relationships modelled in it, is subjective to a certain extent, this should not be equated with arbitrariness. In contrast, all the interrelationships examined and the considerations made, which form the basis for the proposed weightings, were presented in a concrete and comprehensible manner.

Since this procedure relies on (human) “chain of thought” reasoning, artificial intelligence is not yet recommendable for use at its current stage of development [35]. However, the RIMEDA procedure could serve as the foundation for scientific discussion with the aim of further developing and refining it and, last but not least, implementing it in an explainable AI-supported, program-controlled process. Since the demand for medical datasets secured against reidentification will increase significantly, if only the volume of datasets required for training various AI applications is considered, assessing the risk is a crucial step in taking appropriate protective measures. Additionally, the RIMEDA procedure not only enables an estimation of the overall risk but also allows the identification of risk-driving characteristics of a medical dataset, thus providing direct indications of countermeasures to be taken.

Finally, the application tests (see additional file behre_rimeda_supplementary_information.pdf) may have underestimated the actual risk due to a lack of information in the published attacks. Conversely, Xia et al. assume a general overestimation of the reidentification risk due to insufficient modelling of the attacker’s abilities or failure to consider missing data records in the sources required for the attack [10, 36].

## Conclusions and future prospects

While an assessment of the reidentification risk of medical data is currently mainly based on the consideration of one or a small number of influencing factors and is thus subject to certain limitations, our proposal offers a holistic view and a statement about the probable extent of this risk. However, due to the fact that the operationalisation of the influencing factors is difficult and the modelling as such is associated with some subjectivity, a risk assessment cannot be carried out without a certain degree of imprecision. Hence, assessing the risk of unwanted deanonymisation of medical data remains challenging and additional efforts are required to overcome the weaknesses of this approach and to be able to perform a comprehensive risk stratification more objectively overall. The further development of this proposal should also include an application study using different data sets and a study on inter-rater reliability.

In the future, interdisciplinary research opportunities with regard to measurement often exist, for example, in the construction and establishment of a system of key figures to enable an objective assessment of an organisation’s risk awareness. Such a system requires the pooling of expertise from the fields of business administration, business informatics and statistics. For example, the development of a (Bayesian) classifier could help improve the estimation of the inference potential of medical data.

In modelling, the entity “metadata” also has great potential to influence the reidentification risk due to pronounced cross-linking. With respect to patient-related data, few studies have been conducted to date, so the significance of metadata warrants further investigation.

The RIMEDA procedure could, in the long term, form the basis for an explainable AI-based prediction model, which may then provide a more exact statement regarding the reidentification risk of medical data.

## Supplementary Information

Below is the link to the electronic supplementary material.

Supplementary Material 1

Supplementary Material 2

## Data Availability

No datasets were generated or analysed during the current study.

## Abbreviations

ACM: Association for Computing Machinery
AI: Artificial Intelligence
ERM: Entitity Relationship Model
HIPAA: Health Insurance Portability and Accountability Act (USA)
ICD: International Statistical Classification of Diseases and Related Health Problems
IEEE: Institute of Electrical and Electronics Engineers
IT: Information Technology
OCR: Office for Civil Rights (USA)
RIMEDA: Reidentification risk of MEdical DAta
