# Supplementary material for: Proposal of a procedure to stratify the reidentification risk of medical data: RIMEDA
Source: BMC Med Inform Decis Mak. 2026 Apr 11;26:120. doi: 10.1186/s12911-026-03475-4 (PMC13072523; doi:10.1186/s12911-026-03475-4)
Supplement: Supplementary file 1 — Supplementary Material 1 [file 12911_2026_3475_MOESM1_ESM.pdf]

Supplementary material to

Proposal of a procedure to stratify the re-identification risk of medical data: RIMEDA

Table of Content

Explanation and guidance for application 3
1. Definitions and concepts 3
1.1. Data model 3
1.2. Threat model 3
1.3. Anonymity and pseudonymity 6
1.4. Formal anonymity criteria 9
1.5. Metrics for assessing the loss of information 10
2. Modelling risk factors 11
2.1. The data perspective 11
2.2. The knowledge perspective 13
2.3. The attacker perspective 15
2.4. The technical/organisational perspective 16
3. Entity relationship models of the risk factors 18
3.1. Interrelationships of the entities of the data perspective 18
3.2. Interrelationships of the entities of the knowledge perspective 22
3.3. Interrelationships of the entities of the attacker perspective 27
3.4. Interrelationships of the entities of the technical/organisational perspective 30
4. Influence Matrix 34
4.1. Risk assessment 35
Use of the results and recommendations for action 40
Reduction of uniqueness 41
Increasing the similarity 41
Dealing with vulnerable quasi-identifiers 41
Handling of metadata 41
Dealing with data inference 42
The problem of information loss 42
Creating awareness 42
Quality of methods 43
Data security 43
Dealing with potential attackers 43
Evaluating the procedure and determining its method quality 45
Application and evaluation 46
First application test 46
Second application test 51
Meaning of the results of the application tests 56
References 57

Explanation and guidance for application

1. Definitions and concepts

First, a data model is presented in order to bundle concepts that are linked in the context of personal data and data protection. A threat model is then described where terms are introduced that are related to a possible threat to the data model. Complementary to these explanations, established options are then illustrated, the aim of which is to mitigate or avoid the threats and thus maintain data protection.

1.1. Data model

In Article 4 of the European GDPR personal data are defined as "any information relating to an identified or identifiable natural person (‘data subject’); an identifiable natural person is one who can be identified, directly or indirectly, in particular by reference to an identifier such as a name, an identification number, location data, an online identifier or to one or more factors specific to the physical, physiological, genetic, mental, economic, cultural or social identity of that natural person." [1]. Personal data are generally classified into the two overarching categories of identifying and non-identifying data [2,3]. Identifying data can be further divided into the two subgroups “Direct Identifier”, e.g. social security number, and “Quasi Identifier” which, considered on its own, does not permit identification, but in combination with each other and/or in combination with other information, distinguishes a defined person and thus makes him or her identifiable [4]. Non-identifying data can also be split into two further categories, firstly “Sensitive Data” as attributes that an attacker would like to assign to a specific person, e.g. ICD-10 codes, and secondly "Non-Identifier" representing data that is either of no interest to a potential attacker or is not suitable for re-identification [5]. The Health Insurance Portability and Accountability Act of 1996 (HIPAA) is a U.S. law that regulates the collection and handling of "protected health information" or PHI. It designates 18 directly identifiable characteristics that must be removed from medical data records in order for them to be legally ("safe harbour de-identification") [6].

In the context of this work, only data that is available in tabular or relational form is considered.

1.2. Threat model

A risk can be defined as a "combination of the probability of occurrence of an adverse event and the resulting level of damage" [7]. In the context of data protection, the amount of damage results on the one hand from the sanctions laid down in the GDPR, which can be very severe depending on the severity of the breach. In addition, there is a further level of damage in the form of loss of trust in

the organisation managing the data, which, like the intangible damage to the individual affected, is difficult to quantify but cannot be neglected, particularly in the medical context [8,9].

Medical or patient-related health data represent a subset of personal data with regard to the often directly identifying characteristics (e.g., hereditary information), whereby a definite delimitation is difficult. To make the rather abstract concept of a "data privacy risk" more tangible, the construction of a "threat model" lends itself to better illustration. Personal data are viewed in terms of potential attacks and the objectives pursued. Basically, two or three possible threats to personal data are distinguished [10]:

a) *Identity Disclosure:* An explicit reference to a person can be established. This means that a specific data record is assigned to a specific person.
b) *Attribute Disclosure:* Through an appropriate analysis of the data records, further or additional information about a specific person becomes known.
c) *Membership Disclosure:* A special case in the context of attribute disclosure, whereby a particular individual can be shown to be a member of a data collection under investigation [11]. This may be, for example, participation in an HIV study, with potentially negative consequences for the individual if disclosed. The erroneous assignment of a person to a corresponding group also represents a risk for the person in question [12].

This work focuses on the reconstruction of a personal identity, as it is the most relevant in terms of both the current legal situation and the corresponding literature [13].

Identity and attribute disclosure are the target of so-called re-identification attacks (synonym: de-anonymisation attacks). A total of four conceivable situations can be distinguished in which such attacks can occur [2]:

a) *Targeted attacks:* An attacker deliberately and specifically attempts to re-identify one or more data records.
b) *Unintentional Reveals:* An individual or their dataset is accidentally or unintentionally re-identified by a third party because identifying characteristics of that very person are known to the attacker.
c) *"Data leaks":* A threat situation for the data occurs in the context of a theft, loss of a data carrier or similar incidents (so-called "data breaches"). Studies by the U.S. HIPAA Journal show that there has been a steady increase in the number of data breaches over the past years [14]. In this respect, this scenario is becoming increasingly important.

d) *Open data [15]:* Data collections of different origins are made publicly available and can thus be used by all interested parties without further control with regard to processing and purpose limitation.

The National Institute of Standards and Technology (NIST) characterises the following five reasons for a person's motivation for a re-identification attack against a particular dataset [16]:

a) *Verification of the quality of a de-identification measure:* A commissioned attack on an anonymised or pseudonymised dataset to check whether a personal reference can be reconstructed or not.
b) *Reputation:* Increased reputation for a professionally carried out re-identification attempt. (i.e. in the context of scientific research) published in a relevant journal on the basis of a noteworthy methodology.
c) *Embarrassing or exposing the data-collecting agency:* Due to the obligation to handle personal data securely, a successful re-identification attack represents a demonstration of inadequate handling of this data. This is intended to draw attention to inadequate data protection measures or even to damage the affected entity through the implicit loss of trust.
d) *Generating a direct benefit for the de-anonymising party:* The Cambridge Analytica scandal, which came to light in connection with the 2016 U.S. presidential election, demonstrates as a concrete example how data from different sources was combined with personal data to influence voting decisions (so-called microtargeting) and thus generate a benefit for a specific position [17].
e) *Extortion:* An attacker can threaten to publish, for example, stigmatising diagnoses such as sexually transmitted diseases with extortionate intent if he has succeeded in a re-identification attack on corresponding data material.

For a re-identification attempt, at least two datasets must exist: the dataset to be examined and additionally a dataset with identifying features [18]. In the linking process, these datasets are unified, and the resulting overlap of corresponding quasi-identifiers can then be used for re-identification. The more additional data is available, the more likely a successful re-identification attempt is. For the implementation of linking attacks, a software library, the Python Record Linkage Toolkit developed by Jonathan de Bruin (Utrecht University), has been available since 2019 [19]. The use and effectiveness of linking methods for re-identification of medical data has been studied several times, examples include Dusetzina et al. and Hejblum et al. [20,21].

Fung et al. follow the principle threats to personal data described above and further distinguish re-identification attacks into:

a) *Record Linkage:* This attack model aims at identity disclosure. The value of a quasi-identifier marks a small group of records in a shared table. If the victim's quasi-identifier matches that of the group, there is a risk of being linked to the group. Using additional knowledge, the attacker can uniquely identify the victim's record from the group.

b) *Attribute Linkage:* The goal is attribute disclosure, i.e., the assignment of a sensitive attribute to a specific person. A number of identical quasi-identifier in two tables were every record in one table has a counterpart in the other table allows a joining of the tables and thus disclosing of the associated sensitive attributes.

c) *Table Linkage*: an attempt is made to prove membership in a data collection. These attacks are very similar to attribute linkage in their execution. The difference is that a defined combination of attributes is used to prove a victim's membership, rather than the existence of a single attribute [22].

In addition to these re-identification approaches, a number of other methods have been described in the literature that aim at anonymised or pseudonymised data. Ultimately, these approaches are usually of a "preparatory" nature, because they usually attempt to prepare the ground for subsequent linkage attacks. Examples of such methods include de-noising and tracker attacks [23,24].

1.3. Anonymity and pseudonymity

The difference between pseudonymised and anonymised data is that pseudonymisation generally allows for the possibility of attribution, or at least makes it conceivable. In the case of anonymised data, such an assignment option no longer exists on a permanent basis. It is difficult to generate completely anonymised data, since it cannot be ruled out with certainty that a reference to a person can be reconstructed by merging different datasets and subsequently using Big Data applications [25]. Because of this difficulty, the approaches described below aim to create so-called de facto anonymity. This means that data can only be assigned to the respective person with a disproportionately large effort in terms of time, costs and manpower.

To implement the protection of personal data, various approaches are available that remove or modify potentially identifying characteristics or combinations of characteristics:

a) identifying attributes are suppressed or removed

b) data values are intentionally changed

c) the association between attribute and person within a dataset is broken

d) data is artificially created based on the original data

In principle, these methods can only be applied to tabulated or tabularly recordable data. In the case of signal data such as ECG data, radiological data such as MRI images, or audio and video data, the measures described in the following are not useful.

1.3.1. Removing identifying or prominent features

Suppression is the simplest method of removing or reducing the personal reference from a dataset. Record suppression completely removes a dataset to be protected, value suppression removes all instances of a defined data attribute, and cell or local suppression removes only selected instances [26–28]. Top coding eliminates all datasets or attributes whose instances are above a predefined threshold, while bottom coding eliminates all those below a threshold [29]. However, this method is not suitable for epidemiological questions or potentially needed contacts, e.g., in the context of clinical trials [30]. In addition, the removal of these characteristics alone is usually not sufficient to ensure complete anonymisation.

1.3.2. Randomisation

The basic idea of randomisation is to replace existing data values in a dataset with random values in order to make the personal reference unrecognisable. However, for further scientific use of the data, it is necessary that the statistical properties of the dataset are preserved [31]. Different approaches to randomisation are known, often also referred to as perturbation. The following selection is not to be understood as exhaustive:

a) *Shuffling/swapping:* Data values of one person are swapped as randomly as possible with the values of another person in the dataset to be examined [32]. In this way, the relevant values of the dataset are retained, but the respective person reference is removed.

b) *Use of hash functions:* Hash functions are cryptographic algorithms that map strings of arbitrary length to a fixed-length hash value, making them well suited for creating pseudonyms. Cryptographically suitable hash functions exhibit one-way properties and are collision resistant. Despite these properties, successful attacks on hash functions have been described, so careful selection of the hash algorithm to be used is essential for sufficient pseudonymisation [33].

c) *Noise addition:* Sensitive parts of the dataset are deliberately overlaid with a random measurement error [34]. This technique is only suitable for numerical values and has been criticised since there are described attacks in which the data could be recovered by special filtering techniques [35]. It can also significantly reduce the usefulness of the data for further analysis.

1.3.3. Generalisation, Aggregation

In the context of a generalisation or aggregation, data are combined into hierarchically organised categories with regard to a common feature. characteristics to hierarchically organised categories. This "coarsening" achieves a (gradual) reduction in data specificity, so that a certain data record can no longer be precisely assigned to an individual person [30].

A distinction is made between methods that generalise either all (global recoding) or specific (local recoding) instances of an attribute in a dataset. Several generalisation schemes are described for this purpose [22]:

a) *Full-Domain Generalisation*: All values of a data attribute are generalised at the same level. Conceivable generalisations can, for example, consist of converting explicit age data into intervals or subsuming concrete diagnoses under a generic term [30,36,37].

b) *Subtree Generalisation:* All child nodes of a specific parent node are generalised. Example: Occupations in a dataset are grouped into categories, all manual occupations are generalised, while administrative occupations are not [26].

c) *Sibling Generalisation:* This scheme differs from the subtree scheme in that only specific child nodes of a parent node are generalised. Example: "plumber" is replaced by the generic term "craftsman", but "carpenter" is not [37].

d) *Cell Generalisation:* This approach allows the generalisation of selected single values and therefore represents an example of local recoding [38].

e) *Multidimensional generalisation:* In this schema, all conceivable combinations of data attributes are possible on different levels of a generalisation hierarchy. Referring to the previous examples, multidimensional generalisation allows for statements such as "Occupation: plumber; age: 50-60" or "Occupation: craftsman; age: 52", etc. [39].

1.3.4. Anatomy

Anatomy is a method which, unlike generalisation or suppression, does not change the identifying characteristics or the sensitive attributes themselves. It is proposed to store (quasi-) identifiers and the sensitive data to be protected separately in separate tables and to logically link them via a common group ID. In this way, the association of the identifier with the respective sensitive attribute is removed or "softened" [40].

1.3.5. Slicing

Slicing also refrains from altering the datasets [41]. Instead, the data is divided into n horizontal and m vertical partitions so that a grid of $n \times m$ partial relations is created, which are then permuted. The

data is subsequently reassembled into a new relation that no longer contains the original personal reference [42].

1.3.6. Synthetic Data Generation

Based on statistical characteristics of the original data, artificial datasets are generated with the help of mathematical models. These synthetic data then form a subset of the original data and can be used for further processing due to their statistical similarity to the original data [43].

1.4. Formal anonymity criteria

In the following, common formalised criteria by which the achieved "degree of anonymity" can be assessed after application of the presented methods are introduced. These anonymity criteria can also be used as instruments for anonymisation, but here they are to be considered as "quality measures". The ARX-Data Anonymisation Tool13 is an open-source software solution for determining the anonymity measures and also the loss of information [44].

1.4.1. k-Anonymity

The criterion or model proposed by Samarati and Sweeney is widely used in the scientific literature and defines a table as *k*-anonymous if each row with respect to the quasi-identifiers occurs exactly *k* times [45]. In order to achieve *k*-anonymity, identifying characteristics of a dataset are first determined, then generalised and aggregated to so-called "equivalence classes". A diagnosis can then no longer be assigned to a specific person, but only to a specific "equivalence class" of at least k generalised entries. *k*-anonymity thus assesses the protection against identity disclosure. With a higher *k* value, stronger anonymity is assumed. In the (bio)medical field, a value of *k*=5 is often aimed for; values between *k*=3 and *k*=25 are considered normal [46].

1.4.2. ℓ-Diversity

Since *k*-anonymity involves risks with regard to the disclosure of sensitive attributes (e.g. homogeneity attack, background and knowledge attack), another anonymity criterion was developed in the form of ℓ-diversity [47]. This is an attempt to ensure that a sufficiently large number of sensitive attributes are present in an equivalence class, thus preventing the reconstruction of a person reference . Hence, Machanavajjhala et al. define an equivalence class as ℓ-diverse “if it contains at least ℓ ‘well represented’ values for the sensitive attribute” and a table is ℓ-diverse if all equivalence classes are ℓ-diverse [47].

However, with the so-called skewness and similarity attacks, limitations of the ℓ-diversity were revealed [48].

1.4.3. t-Closeness

To overcome the weaknesses of ℓ-diversity in preventing attribute disclosure, Li et al. proposed a further anonymity criterion, *t*-closeness, as a refinement of the two previous anonymity criteria [48]. In order to fend off attacks on ℓ-diversity, the distribution of the attributes to be protected within the equivalence classes should ideally differ as little as possible from the overall distribution of the attributes. *t*-closeness aims to quantify the knowledge that an attacker can draw from the distribution of attribute values in relation to the overall distribution into a value *t* that correlates negatively with the strength of anonymisation.

The formal definition of *t*-closeness by Li et al. states that an equivalence class is *t*-close if the distance of the distribution of a sensitive attribute does not deviate by more than *t* from the distribution of the attribute in the entire table. A table is *t*-close if all equivalence classes are *t*-close [48]. To determine *t*, the distance of the attribute distribution between the considered equivalence classes and the overall distribution, a distance measurement method called Earth Mover's Distance (EMD) is proposed [48,49].

1.4.4. Differential Privacy

Differential privacy is intended to prevent re-identification attempts by means of background knowledge (linkage attacks) [50]. A statistical comparison between a dataset with a defined individual and a dataset without this individual should show no significant differences after the application of anonymisation methods. This also disguises the membership of an individual in a dataset (so-called membership disclosure) [22]. To implement this, the use of a random factor or "noise" is proposed, which can be sampled from a Laplace distribution, for example [51].

1.5. Metrics for assessing the loss of information

The application of anonymisation methods leads to a more or less pronounced loss of information content and thus of data usability. In order to be able to capture this loss, several methods or metrics have been proposed. In the following, a short description of the most common methods used to quantify the information loss of generalisation according to Kohlmayer will be given [52].

a) *Height metric*: There is an inverse relationship between the information content of a dataset and the height of the generalisation hierarchy, i.e., the more generalised the data, the more information is lost. The loss of information is expressed by the sum of the height of the generalisation hierarchies of all data attributes [45].
b) *Precision metric*: Similar to the height metric, the heights of the generalisation hierarchies are included, with the respective hierarchy being normalised with its maximum possible height [36].

c) *Loss metric*: Like the first two approaches, the loss metric also uses the height of the generalisation hierarchy, examining the proportion to which the transformed data represent the original data. The proportions are summed for all attributes [53].

d) *Average equivalence class size*: calculating the average value of the equivalence class size of the data collection after generalisation measures have been performed can be used as a measure of information loss [39].

e) *Discernibility metric*: This metric also focuses on the equivalence classes after a generalisation and "punishes" indistinguishable tuples. The higher the overall "penalty" on the entire dataset, the higher the observed information loss [26].

f) *Entropy*: In information theory entropy is a measure of information content. Therefore, the entropy measure can be used as an indicator for the loss of information after the application of an anonymisation procedure [54].

2. Modelling risk factors

According to the results of the literature review, influencing factors - or entities - could be assigned to four overarching entity groups, referred to below as perspectives: The data, the knowledge, the attacker, and the technical/organisational perspective.

2.1. The data perspective

The data perspective firstly includes all those entities that can be derived from the data using mathematical methods and thus represent precise metrics. In addition, quasi-identifier combinations considered to be particularly risky are grouped together to form the entity "Vulnerable Quasi-Identifier".

The degree of uniqueness U of a data collection is usually calculated as the number of unique records f with unique attribute combinations relative to the entire collection F under consideration: $U = (f\ /\ F) * 100\%$. The more unique records exist, the more likely it is to induce a re-identification of one of these records by means of a linking attack. Methods for determining similarity can be used to analyse the data collection under review for similar combinations of characteristics, cluster analyses are well suited for this purpose [55]. If two identical datasets are considered to be maximally similar, it can be deduced that less pronounced similarity structures indicate differently or even uniquely configured datasets. From this, the degree of re-identification risk can be estimated.

Table 1 provides an overview of the entities in the data perspective.

Table 1. Data perspective (supertype)

Entity	Attribute	Domain	Weighting
Uniqueness	Proportion of total number of records	[0%, 100%]	0 - 3
Similarity	Number of similar records	[0%, 100%]	0 - 3
Vulnerable Quasi-Identifier	Length of stay	[not] included in the dataset	0 - 3
	Treating physician		
	Diagnosis		
	Date of admission		
	Date of birth		
	Sex		
	Zip code		

A commonly used measure in the literature for the number of unique record sets, which is supposed to represent a just acceptable risk, is between 5% and 20% of the total amount of data considered [56], so that based on this the following weighting is suggested:

- 0 points No uniquely configured records available
- 1 point 1 - <5% uniquely configured records, acceptable
- 2 points 5 - ≤20% uniquely configured records, still acceptable
- 3 points >20% uniquely configured records, unacceptable

To the best of our knowledge, thresholds for similarity between datasets have not yet been described in the scientific literature. Based on the inverse relationship between the risk potential for de-anonymisation and the degree of similarity structures, it is proposed to use the proportion of datasets which can be clustered as a measure for weighting. The thresholds for the number of unique records are used for this purpose:

- 0 points >20% of the datasets aggregated to clusters, strong similarity
- 1 point 5 - ≤20% of records aggregated into clusters, decreasing similarity
- 2 points 1 - <5% of records aggregated into clusters, barely similar
- 3 points no similarities detectable, unambiguous

The characteristics of the entity "Vulnerable Quasi-Identifier" result from the elaborations of Lee and Lee [57]. Since the risk potential of these characteristics increases with their number, the following weighting is proposed accordingly:

- 0 points no vulnerable quasi-identifiers present
- 1 point maximum one vulnerable quasi-identifier present
- 2 points up to 2 vulnerable quasi-identifiers present
- 3 points ≥3 vulnerable quasi-identifiers present

2.2. The knowledge perspective

The level of additional knowledge required to restore a personal reference, is called the knowledge perspective. In contrast to the data perspective, the entities of this supertype cannot be represented by a specific function, but are subject to a certain fuzziness with regard to their respective attributes and domains. The entities "external data sources", "data inference" and "background knowledge/context" extracted from the reviewed literature are classified to the knowledge perspective. Furthermore, it is proposed to add the entity "metadata". Metadata is data that describes selected aspects of other data. Metadata is particularly relevant in the area of medical data [58]. The potential of metadata in terms of data protection risks is highly significant and should therefore not be neglected in a risk assessment [59].

Different types of metadata are distinguished [60]:

- administrative: information about author, rights, tracking, etc.,
- descriptive: describing a dataset, e.g. by means of title information, release dates or similar,
- preservation data: information about the preservation of data integrity, such as checksums,
- provenance data: information concerning the origin of the data,
- usage data: provides information about the use of the data, such as citations,
- technical/structural: representation of technical parameters such as file size, timestamps, etc.

There are currently no known publications on re-identification risks due to metadata in medical data, but the risk potential of metadata with regard to data protection has been obvious since Edward Snowden's revelations on secret service activities and should therefore not be neglected in a risk assessment [61].

The entities of the knowledge perspective are basically characterised by three attributes:

1. *Type*: Suitable external data sources include electoral rolls, death registers, accident statistics or, increasingly, social media data. Based on the data attributes contained, the data collection to be attacked or assessed can provide indications as to which external source could be promising in terms of de-anonymisation. For example, if death data are included, obituaries may be a suitable data source; persons affected by a rare disease may seek support in a specialised forum.

Background and contextual information can be easily obtained by means of social engineering, metadata can be divided into several categories, as briefly introduced above.

2. *Suitability*: A data source is suitable for re-identification if it contains directly identifying characteristics and combinations of characteristics that overlap with the dataset to be attacked. It can thus be considered "suitable" or "unsuitable" in binary terms. Additionally, the high potential for correlating information of metadata must absolutely be included in the assessment of suitability.
3. *Availability*: An additional data source must be available at all in terms of financial, technical and legal aspects in order to be worthwhile for a re-identification attempt. Stolen data or information obtained by means of social engineering must be included in the consideration.

There is no known statistical measure for data inference. It is basically up to the experience (and creativity) of the assessor to judge whether further information can be derived from a dataset.
Table 2 provides an overview of the entities in the knowledge perspective.

Table 2. Knowledge perspective (supertype)

Entity	Attribute	Domain	Weighting
External data sources	Type	undetermined	1 - 3
	Suitability	[unsuitable; suitable]	
	Availability	[low; medium; high]	
Background / contextual information	Type	undetermined	1 - 3
	Suitability	[unsuitable; suitable]	
	Availability	[low; medium; high]	
Metadata	Type	see “metadata”	0 - 3
	Suitability	[unsuitable; suitable]	
	Availability	[[low; medium; high]	
Data inference	Potential	[low; medium; high]	1 - 3

First, the data sources under consideration must be reviewed for suitability, and then an assessment of the resource under consideration can be made in terms of its technical, financial, and legal availability. The key question is: What effort is involved in acquiring the external knowledge? The easier it is to acquire additional information, the higher the probability of unwanted re-identification. Accordingly, a score of "0" implies the complete absence of additional knowledge and is not realistically conceivable, with an exception for metadata, since this is technically manageable:

- 3 points high availability; e.g. through publicly available information
- 2 point medium availability; such as information available for purchase
- 1 point low availability; e.g. information that can only be obtained illegally
- 0 points suppressed (metadata)

As already mentioned, the experience of the analyst is of decisive importance for the assessment of data inference, no objective yardstick can be found for weighting here. In the context of medical data, however, experience has shown that the potential for data inference is high. This is particularly true if the data are reviewed by a person who is medical expert [62]. In this respect, a score of "3" is suggested for a potential assessed as high, and lower values for corresponding lower classifications, with a value of "0" not being reached.

2.3. The attacker perspective

The attacker perspective assesses the influence of a potential attacker on the re-identification risk. In this supertype, the entities "motivation," "skills," and "financial/time/technical resources" are combined. For example, one can assume that an attacker with a secret service background should be judged as more potent with respect to these entities than, say, a nosy neighbour. One study showed that, with few exceptions, most re-identification attacks were carried out exclusively by attackers with a scientific background (and thus corresponding methodological knowledge) [63]. Nevertheless, this entity cannot be modelled precisely, since it can only be conjectured who may have an interest in reconstructing a person reference and what the "qualities" of this person(s) are.

This perspective is largely based on the assessment of the threat situation by the responsible body, the more "pessimistic" this situation is viewed, the more weight should be given to this perspective.

Table 3 gives an overview of the entities in the attacker perspective.

Table 3. Attacker perspective (supertype)

Entity	Attribute	Domain	Weighting
Motivation	Degree	[low; medium; high]	1 - 3
Skills	Degree	[low; medium; high]	1 - 3
Resources	Degree	[low; medium; high]	1 - 3

The entities of the attacker perspective should be simply weighted in correlation with the suspected degree:

- 3 points high degree of the entity
- 2 point medium degree of the entity
- 1 point weak degree of the entity

A highly motivated attacker with strongly trained statistical skills and quasi unlimited resources (e.g. the above mentioned secret service background) would thus receive the maximum score of 3+3+3=9 possible points. The point value "0" is not assigned here because it unrealistically implies that there is no threat.

2.4. The technical/organisational perspective

Finally, environmental topics are unified in a technical/organisational perspective. Only one mention of this topic was found in the literature[64], but statistics from the Health Insurance Portability and Accountability Act (HIPAA) Journal on data breaches, among others, show that this perspective should not be neglected [14]. Therefore, the entities "awareness", "quality of methods", and "data security" are subsumed here. This supertype has the property of reducing the probability of occurrence of re-identification. In the influence matrix, this effect is later expressed in the form of negative point values.

In the context of this perspective, "awareness" means that the persons responsible in the hospital or institution are fundamentally aware of the data protection risk posed by medical data and that appropriate measures to minimise the risk are sought and implemented. To date, no defined indicators are available for assessing awareness. Although prototype approaches exist in the literature [65], and from some IT security service providers, a specific method has not yet been established. It is problematic that there is no satisfactory definition for the concept of "awareness", making standardised measurement difficult.

It is therefore up to the responsible bodies to make a self-reflective assessment or to seek support from specialised service providers. The awareness model presented by Helisch, for example, which comprises three elements, can be used as a guide [66]:

- *Knowledge*: The responsible level, and also the employees are aware of the causes and effects of data privacy risks; there is a corresponding sensitivity to the issue. Relevant laws and guidelines are known in this context.
- *Will*: The responsible bodies show a concrete intention to act, which manifests itself, for example, in the appointment of a data protection officer with corresponding powers. In this context, data privacy and data security measures are seen as an investment rather than a burden.
- *Capability*: This addresses the organisational level of an institution, including aspects such as an obligation to provide further training, the appropriate design of (business) processes,

the establishment of a data privacy management system, or sanctions for violations of defined guidelines.

To operationalise the entity, the perspectives are assessed in terms of their degree. The more concisely the elements of the awareness model can be depicted, the stronger the existing awareness is rated. A strong awareness is assigned a score of "3", as awareness of the problem decreases, the values drop, and finally, in the case of complete lack of interest, a score of "-3" is assigned, because in this situation a sharp increase in risk is to be expected as a result of deliberate ignoring.
In addition to the totality of tools used to reduce data protection risk, the "methods" entity primarily comprises their quality, by which is meant how well a particular de-identification method protects against a re-identification attack. For example, the exclusive removal of directly identifying features is not sufficient to ensure effective protection against the re-identification of a person.

To classify the quality, the following weighting is proposed using these criteria:

a) The transfer of unprotected raw data is generally unsuitable and represents negligent handling of the data or even a violation of the General Data Protection Regulation (GDPR). Such a practice increases the re-identification risk intentionally and is assessed in the sense of a "penalty" with a point value of "-3".

b) The exclusive removal or masking of direct identifiers does not provide sufficiently effective protection, but can be weighted with a point value of "1" in the sense of a minimal protection strategy ("better than nothing").

c) If the applied measures can fulfil the criteria of *k*-anonymity, a score of "1" is assigned. This value is justified by the fact that k-anonymous data are vulnerable to attack in several ways described [47]. If *k*-anonymity is restricted despite the low protection class, the literature suggests aiming for *k*-values in the interval [5; 25] by generalising the data. The "cell size" concept postulates that *k*-values <5 are not considered sufficiently secure, but that the protective effect of the measures improves with increasing *k*-anonymity [30].

d) If the criteria of l-diversity can be met with the help of the possible protective measures, the score is "2". This value is appropriate because the concept of l-diversity can eliminate the weak points of *k*-anonymity. However, attacks were also described here, so that despite the higher overall level of protection, there is still an increased probability of re-identification.

e) The implementation of the requirements of the t-closeness and differential privacy concepts is considered to be the maximum possible protective effect. To date, no attacks have been described in the scientific literature for either approach. A score of 3 is therefore assigned.

However, the specific implementation often appears difficult due to the peculiarities of medical data [30].

Finally, the entity "data security" will be considered with technical measures to ensure confidentiality, availability, integrity and authenticity. The relevance of this entity arises, for example, from the fact that in 2021 alone, the US authorities were notified of threats to more than 40 million patient records due to security breaches, ransomware, etc. [67].

The ISO 27001 standard is a recognised concept for assessing the data security of an organisation, certification should be sought, especially for large organisations, or at least these guidelines should be followed when operating an IT infrastructure.

A successful certification is assigned a score of "3" in this framework, an orientation to the standards a score of "2"; a "home-grown" action plan is assigned a score of "1". Missing measures are again assigned a score of "-3" due to the avoidable increase in risk.

Table 4 provides an overview of the entities in the technical/organisational perspective.

Table 4. Technical/organisational perspective (supertype)

Entity	Attribute	Domain	Weighting
Awareness	degree	[low; medium; high]	-3; 1 - 3
	Degree of Implementation	[low; medium; high]	
Methods	Type	undetermined	-3; 1 - 3
	Quality	[low; medium; high]	
Data security	Degree of Implementation	[low; medium; high]	-3; 1 - 3

3. Entity relationship models of the risk factors

After the factors influencing the re-identification risk of patient data have been modelled and given a weighting component, the interrelationships and dependencies between the factors are represented in entity relationship (ER) models. In doing so, the individual perspectives are examined for their interactions with the other perspectives in each case. These models serve as the basis for building an influence matrix, which is then used to stratify the re-identification risk.

3.1. Interrelationships of the entities of the data perspective

3.1.1. Uniqueness

The uniqueness as well as similarity of data records are closely linked properties. There is a strong causal relationship between the uniqueness characteristic and the entity of "vulnerable quasi-

identifiers": These identifiers are classified as particularly risk-driving precisely because they are associated with a pronounced uniqueness related to the entire data collection.

Unique datasets are a prerequisite for linkage attacks on the "external data sources", "background knowledge/context", and "metadata" entities of the knowledge perspective. The more unique the dataset is in this context, the more accurate results a corresponding attack can deliver, thus a strong interaction can be assumed. There is also a corresponding interaction with regard to possible data inference, but this depends on the data under consideration and must therefore be regarded as somewhat weaker. For example, an associated diagnosis can often be inferred from a unique combination of medication and procedures.

From an attacker perspective, it can be assumed that knowledge of the possible existence of unique datasets can greatly increase an attacker's motivation to attempt a re-identification attack. Moreover, it drives the development and improvement of an attacker's skills. Finally, identifying unique data consumes attacker resources.

If the risks of unique datasets are known to the responsible persons of an organisation, this may have an increasing effect on their awareness. Analogous to the attacker perspective, knowledge of this entity acts as a driver for the development and improvement of protection approaches. Areas of data security are also affected. In this way, special measures can be initiated that relate to the aspect of "confidentiality", for example.

The relationships described here are depicted in the ER model in Figure 1.

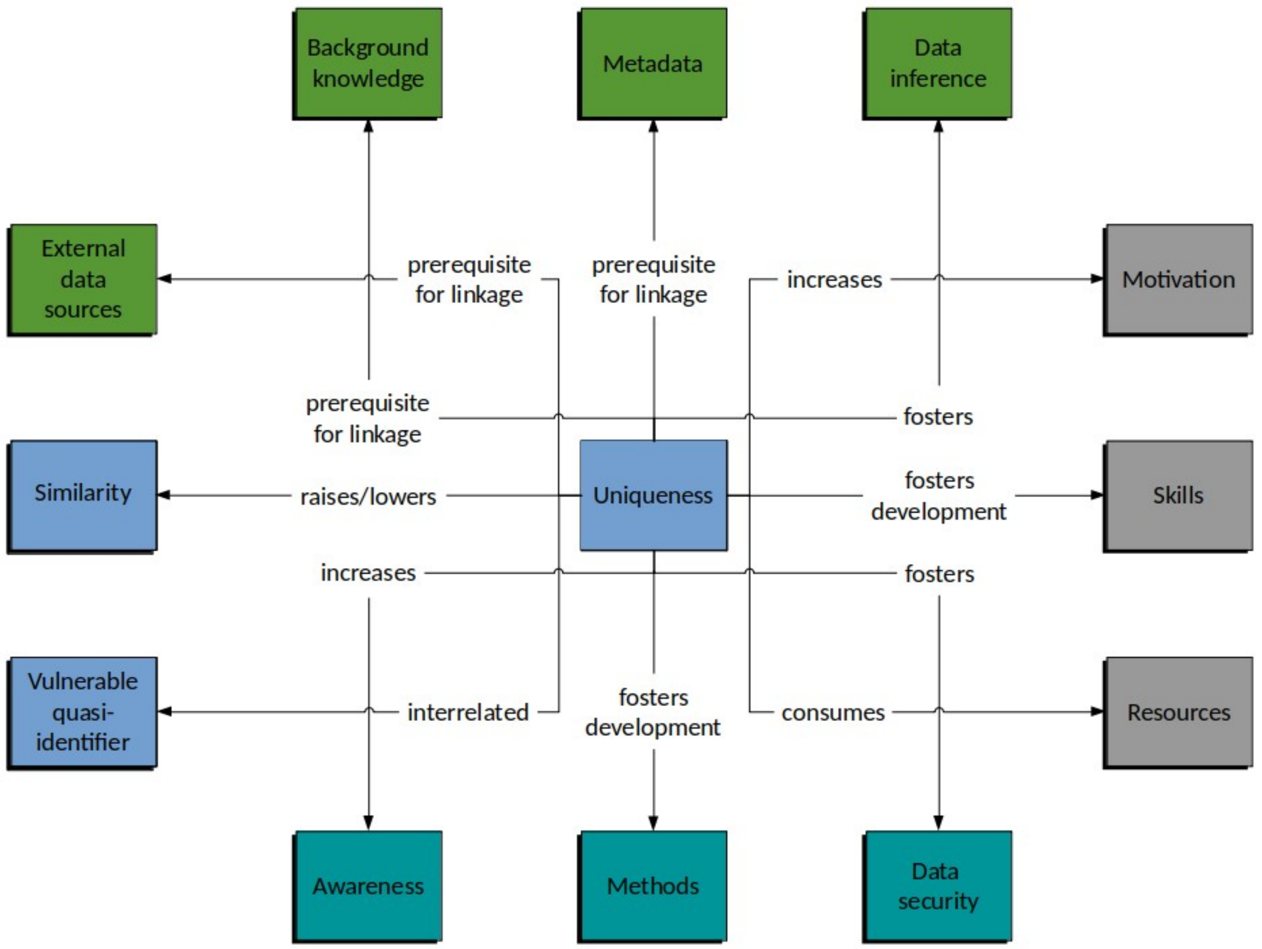

Figure 1. Interrelationships with other entities and their qualitative nature in the case of the entity "Uniqueness".

3.1.2. Similarity

If we look at the interactions of the entity "similarity", relationships can be determined analogously to the entity "uniqueness": The mutual interaction of these two entities has already been described above. There is also an active relationship with the group of vulnerable quasi-identifiers, although this does not manifest itself as strongly as with the previously discussed "uniqueness".

The interactions with the knowledge perspective can also be taken from the above explanations. However, the attribute "similarity" does not provide such a clear-cut possibility for linkage attacks as the "uniqueness" of data, so that this relationship should be classified as weaker overall.

The links with the attacker perspective and the technical/organisational perspective also correspond to those of "uniqueness", albeit to a lesser extent. To summarise, one could say that "similarity" is the "little brother" of "uniqueness".

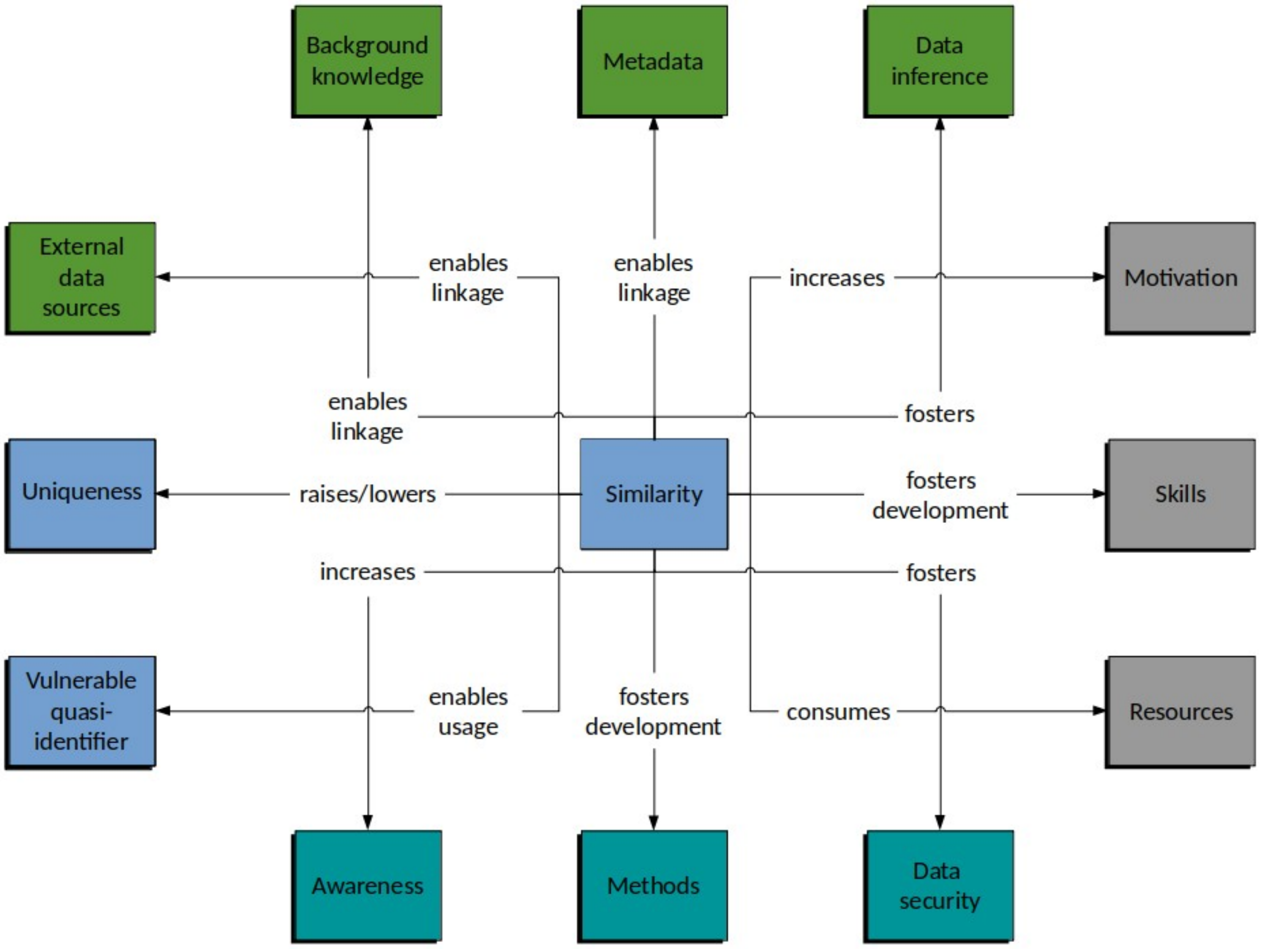

Figure 2. Interrelationships of the entity "Similarity".

3.1.3. Vulnerable quasi-identifier

These combinations of data can create a unique dataset on their own; in combination with other characteristics, the uniqueness of a dataset is then further strengthened, so that the existing interaction can be rated as strong overall. In addition, vulnerable quasi-identifiers can also be used to determine the similarity of datasets; this interaction is considered medium-strong due to the "softer" character of the entity "similarity".

Vulnerable quasi-identifiers interact with the entities of the knowledge perspective because they are used to implement linkage attacks and can also generate correlating information. The more of these identifiers there are in a dataset, the more meaningful the results are. However, as there are only a manageable number of them in total, there are medium-strong interactions. The relationship with the "external data sources" entity is considered strong.

Analogous to the uniqueness and similarity features, vulnerable quasi-identifiers increase the motivation of an attacker and promote his methodological expertise. Of course, analysing them consumes the attacker's resources.

The links with the technical/organisational perspective also correspond to those of the entities already described.

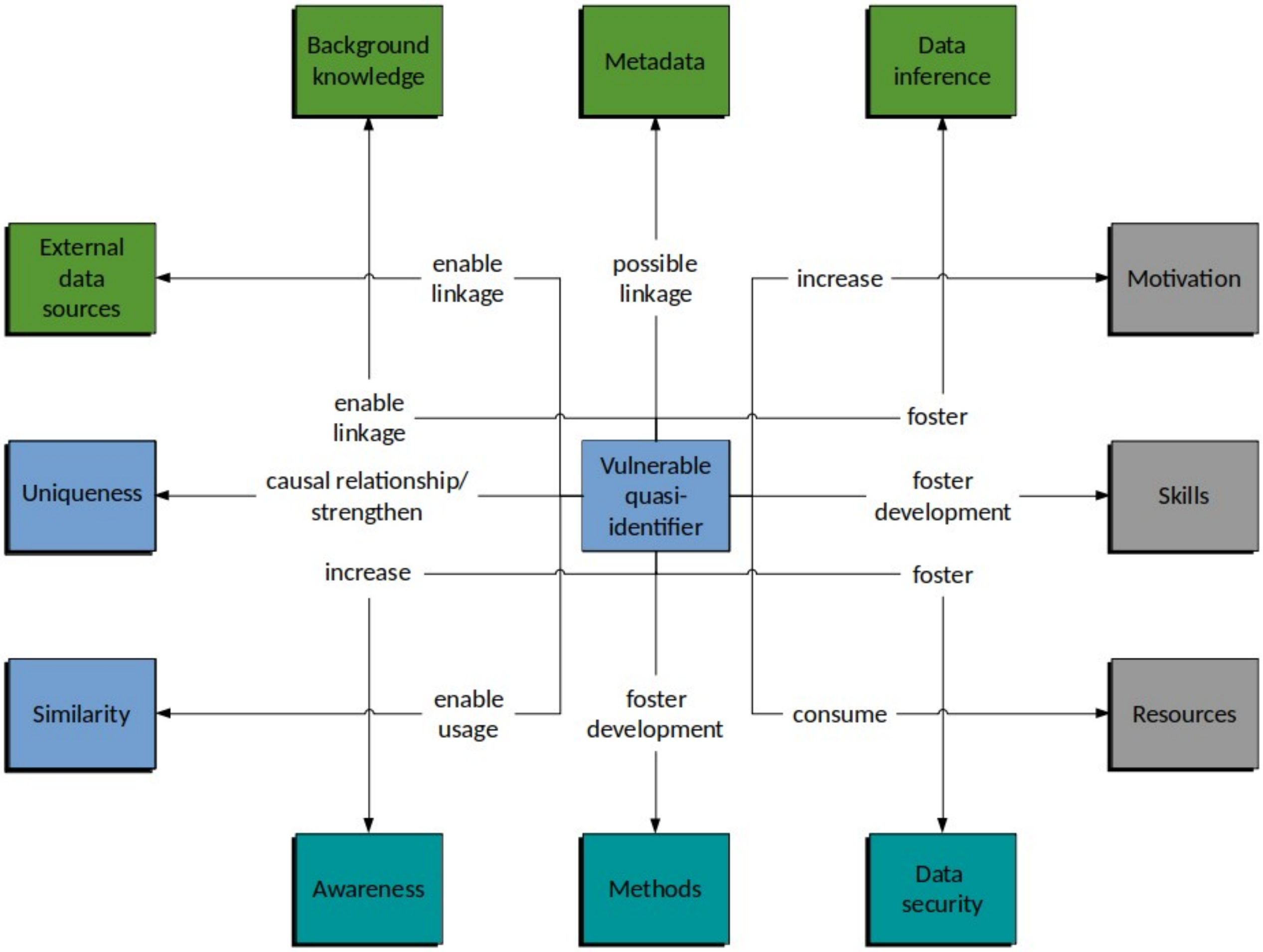

Figure 3. Interrelationships of the entity "Vulnerable quasi-identifier".

3.2. Interrelationships of the entities of the knowledge perspective

3.2.1. External data sources

It is understandable that the existence of external data sources is a basic prerequisite for identifying unique or similar datasets in order to subsequently attempt a linkage attack. It is also clear that vulnerable quasi-identifiers can be extracted from external data sources. In this respect, the existing interaction can be characterised as strong.

The entities of the knowledge perspective interact strongly with each other. For example, it is logical that the factors can be combined with each other or that correlating knowledge can be derived from additional sources.

The availability and knowledge of the potential of external data sources increases the motivation of a potential attacker and thus indirectly also the development and improvement of their skills. More or fewer of the attacker's resources are used to analyse the sources, depending on their availability.

If the risks of external data sources are known to the responsible persons in an organisation, this knowledge should have an increasing effect on their awareness. As with the attacker perspective, knowledge of this entity acts as a driver for the development and improvement of protection approaches. Technical data protection aspects are not influenced by external data sources.

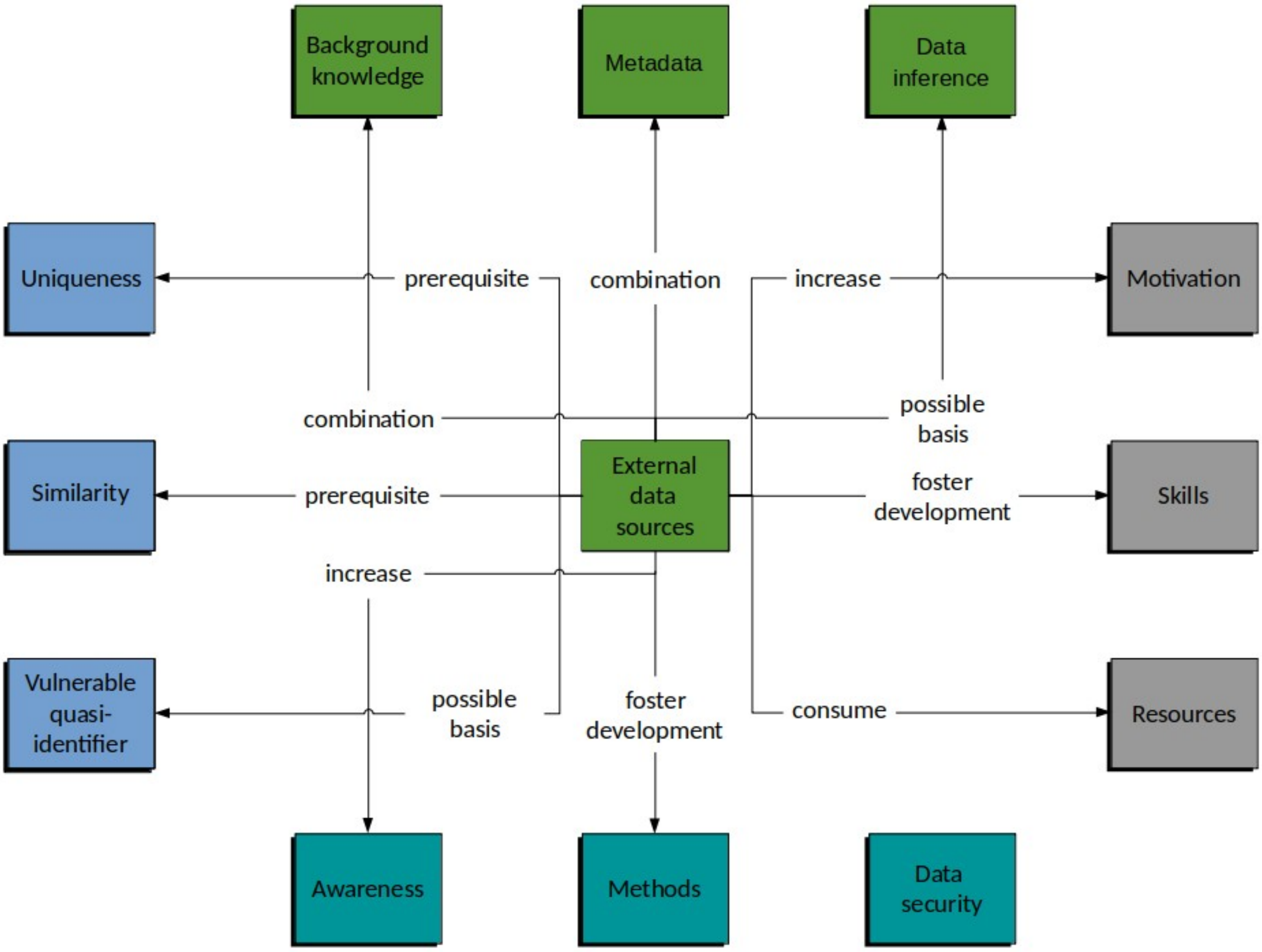

Figure 4. Interrelationships of the entity "External data sources".

3.2.2. Background knowledge

Existing background knowledge or the context in which the re-identification attack is attempted, like external data sources, provide the opportunity to identify unique, similar and/or particularly vulnerable quasi-identifiers. In the context of this work, it is proposed that this effect be considered weaker compared to the external data sources, as the availability and quality of this background knowledge can be highly variable.

Interactions with the entities of the own perspective have already been described above.

Possible interactions with the entities of the attacker's perspective have also been discussed in the previous section.

The explanations given above regarding the technical/organisational perspective can also be applied to the concept of background knowledge.

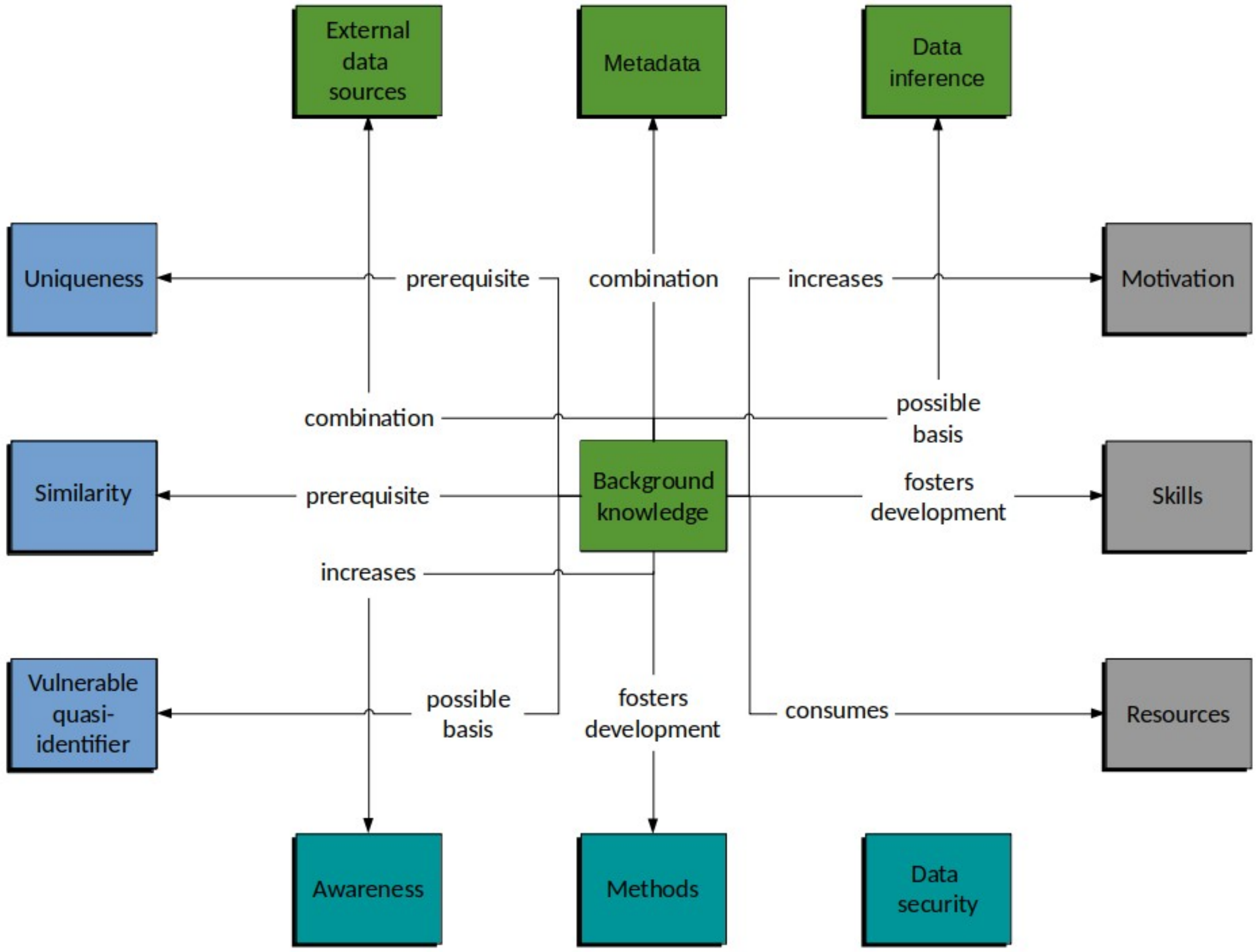

Figure 5. Interrelationships of the entity "Background knowledge".

3.2.3. Metadata

Metadata adds additional information to the actual dataset and can therefore greatly increase its uniqueness. An example of this would be timestamps, which can categorise specific information down to the second, depending on the format. Metadata can also be used to identify similar data records in one or more data collections. To take the above example, it can be concluded from looking at timestamps that the associated data was collected in a narrowly defined and therefore similar time window and therefore presumably belongs to a defined person.

Metadata can also be used to extend vulnerable quasi-identifiers. One obvious possibility, for example, is that a physician's so-called lifelong physician number is stored in the form of metadata together with the diagnoses they have made. All in all, these are strong interactions.

Metadata also interacts strongly with the other entities of the knowledge perspective. Combinations with background knowledge or its supplements are very plausible. In the end, metadata offers a

strong potential for data inference, which is probably even higher than all other relationships with which metadata is linked [68].

With regard to the attacker's perspective, it can also be assumed in this context that the existence of metadata is a strong motivation for a potential attacker. In addition, metadata drives the development and improvement of attack methods because an attacker wants to utilise the potential of this data. As with all the entities already described, analysing metadata also consumes the attacker's resources.

Ideally, knowledge of metadata and the associated risks increases the awareness of the management levels and thus leads to adjustments in the protection tools used. Unlike the knowledge perspective entities discussed so far, metadata can influence data security measures, for example by enforcing consistent storage guidelines or approaches to improve confidentiality (such as strict role and rights management in relation to directory services).

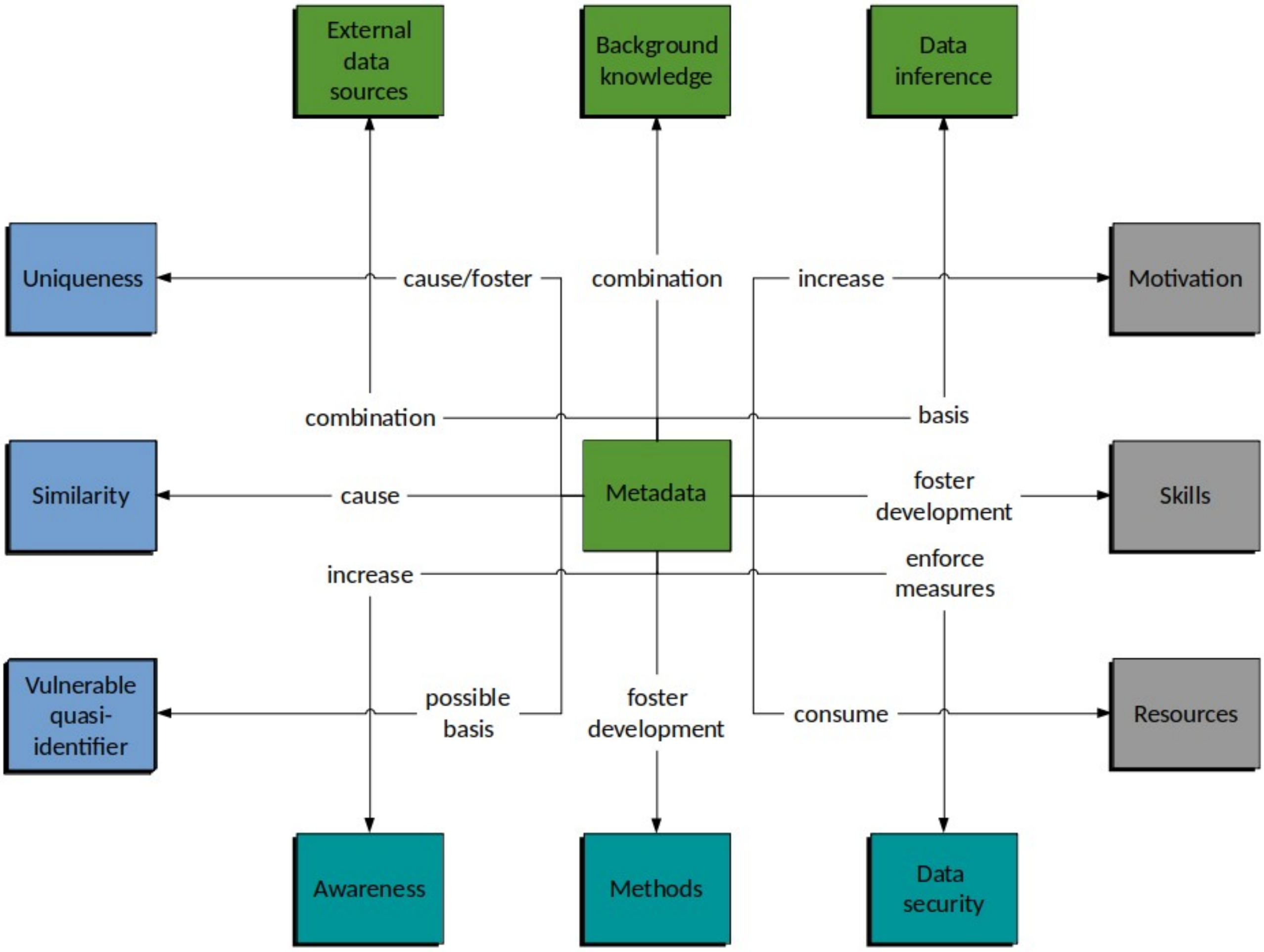

Figure 6. Interrelationships of the entity "Metadata".

3.2.4. Data inference

It is conceivable that data inference contributes to the uniqueness of a dataset. The same applies to the search for similar data records. There is a stronger connection to the vulnerable quasi-identifiers in that, for example - as already explained - a diagnosis can be inferred from information on prescribed medication and procedures performed, which is then available for further processing. In all cases, the assessment depends on the available data.

Data inference and the entities of the knowledge perspective interact directly with each other. This seems immediately obvious: as sources of knowledge, these entities are absolutely necessary for the derivation of correlating data. This interaction must therefore be regarded as strong.

With regard to a potential attacker, it can be assumed that data inference could have an influence on the motivation of a potentially unauthorised person. According to the motto "opportunity makes thieves", it is conceivable that an attacker may decide to attempt re-identification. Data inference also has a favourable effect on an attacker's skills because the person wants to try to benefit from the information derived. Due to the lack of standardised methods, the analysis of data inference takes up more of the attacker's resources than the previously discussed entities.

The interactions of data inference with the entities of the technical/organisational perspective do not differ significantly from those of metadata. Awareness and methods are influenced identically; technical measures, on the other hand, are difficult to implement.

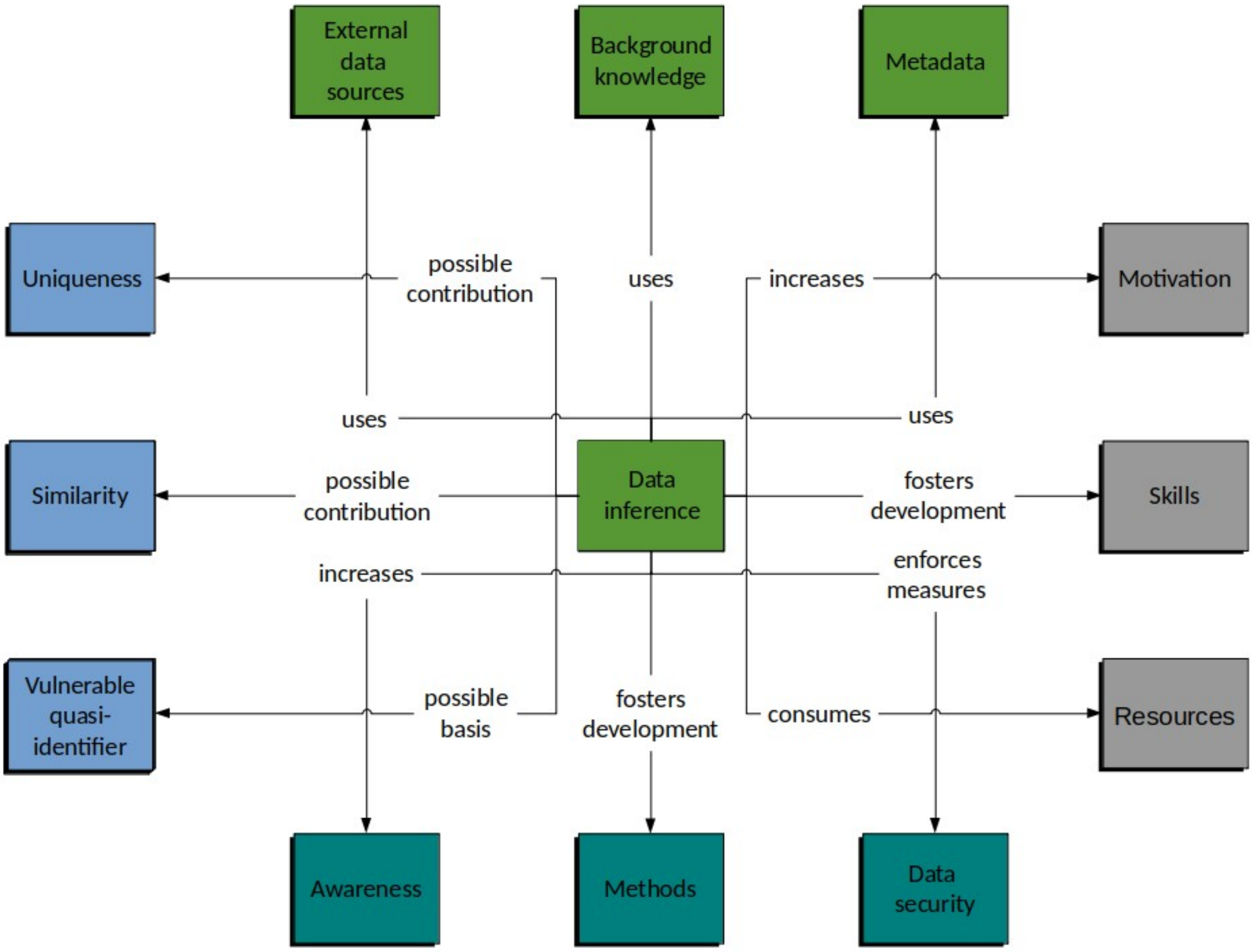

Figure 7. Interrelationships of the entity "Data inference".

3.3. Interrelationships of the entities of the attacker perspective

3.3.1. Motivation

An attacker's drive has no direct effect on the entities of the data and knowledge perspectives. This is understandable insofar as these entities are inherent data properties or data forms in themselves and therefore cannot be influenced by a psychological component or character trait. However, motivation is passively influenced by the knowledge perspective, as already described.

A high level of motivation strongly promotes an attacker's methodological competence and also has an effect on their resources. On the one hand, an attacker is motivated to expand and improve his resources. On the other hand, a high level of motivation also consumes resources because an attacker invests time and financial resources in order to achieve his goal. For this work, the main effect of motivation, which is considered to be strong, is seen in the first assumption.

If an organisation assumes a motivated attacker as part of its risk assessment, at least a minimum level of awareness must be expected. In the knowledge that it is facing a motivated attacker, the

development and improvement of methods to ensure data protection and data security will be accelerated. Overall, these relationships are rated as weak.

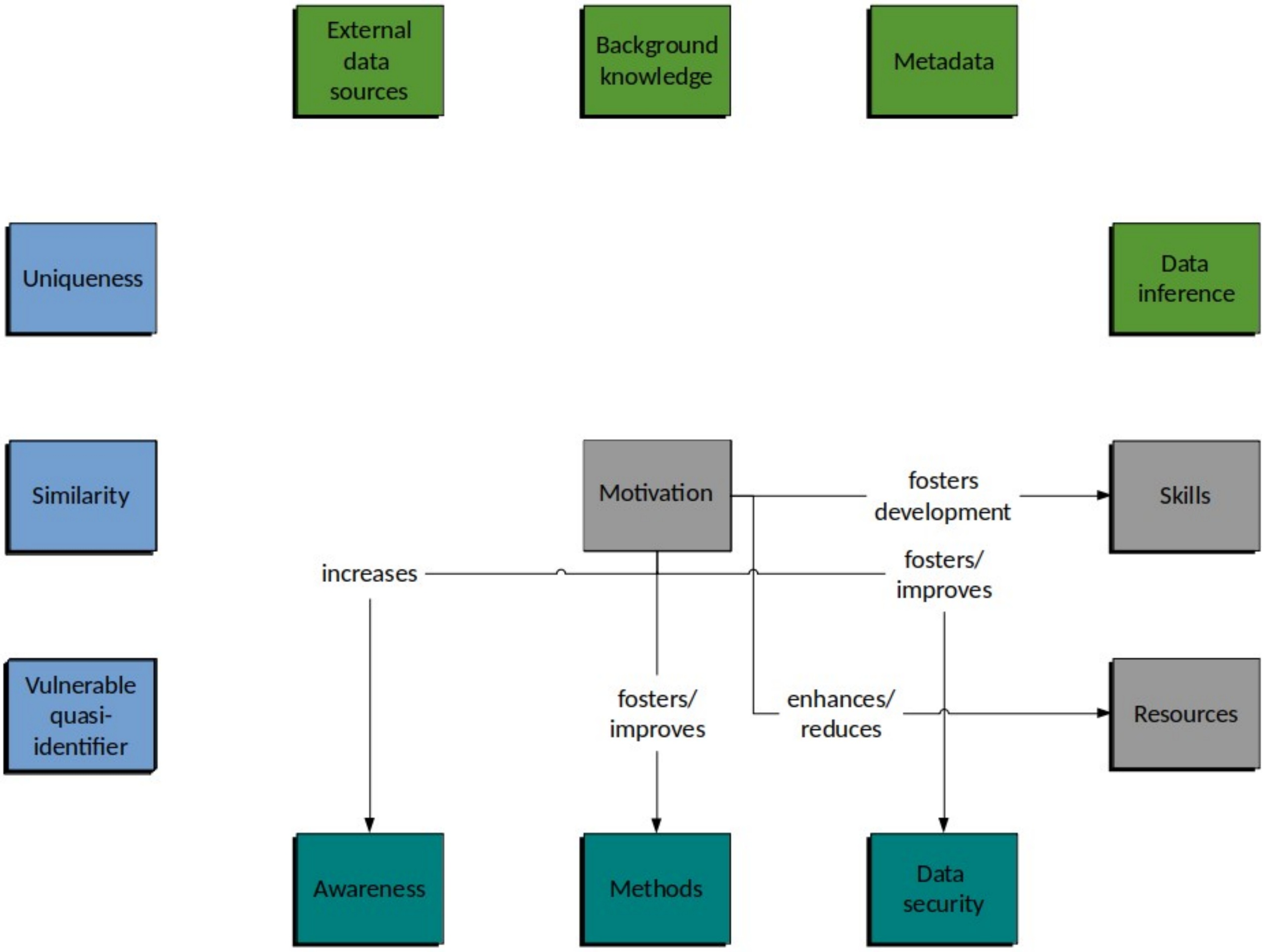

Figure 8. Interrelationships of the entity "Motivation".

3.3.2. Skills

A high level of methodological expertise is required to determine the entities of the data and knowledge perspective and to be able to utilise them in the further course. In this respect, there are strong reciprocal interactions here. Less pronounced skills are required to determine vulnerable quasi-identifiers, as this is more or less a counting problem.

Just as a high level of motivation promotes methodological competence, a high level of skills also increases an attacker's motivation to launch an attack. The use of skills consumes resources; however, it is also conceivable that the attacker uses his knowledge to expand his financial resources, for example. Analogous to the "motivation" entity, the latter assumption is made.

If the organisation is aware of the tools available to an attacker, it can again be assumed that there is a fundamental awareness; this awareness may trigger the development and improvement of protective methods and aspects of data security. Overall, these relationships are rated as weak.

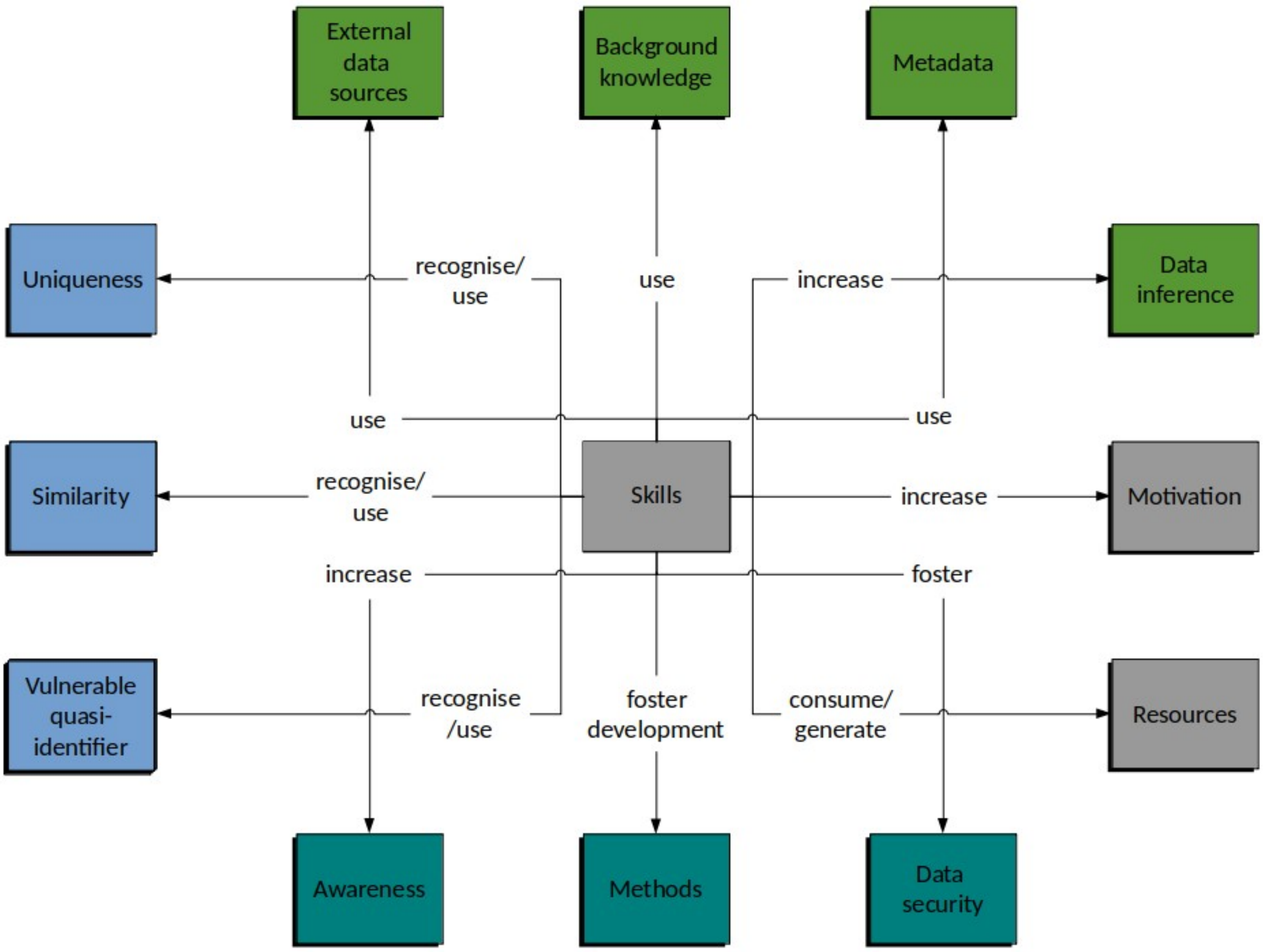

Figure 9. Interrelationships of the entity "Skills".

3.3.3. Resources

The financial and technical resources of an attacker as well as the time available to him are decisive for how intensively he can deal with the entities of the data and knowledge perspective. Despite the fundamental importance of this entity, its overall impact must be considered weaker because even the best resources will not help if the attacker is incompetent.

Sufficient resources are necessary for the development of methodological competence and increase the motivation of the attacker in a comprehensible way. The interaction is therefore considered strong.

If, when assessing the threat situation, an organisation assumes that a financially and technically well-equipped attacker is to be expected, it can be assumed that there is an awareness of the problem. This leads to improved protective measures. The resources of an attacker therefore reactively provoke further developments with regard to the technical/organisational perspective.

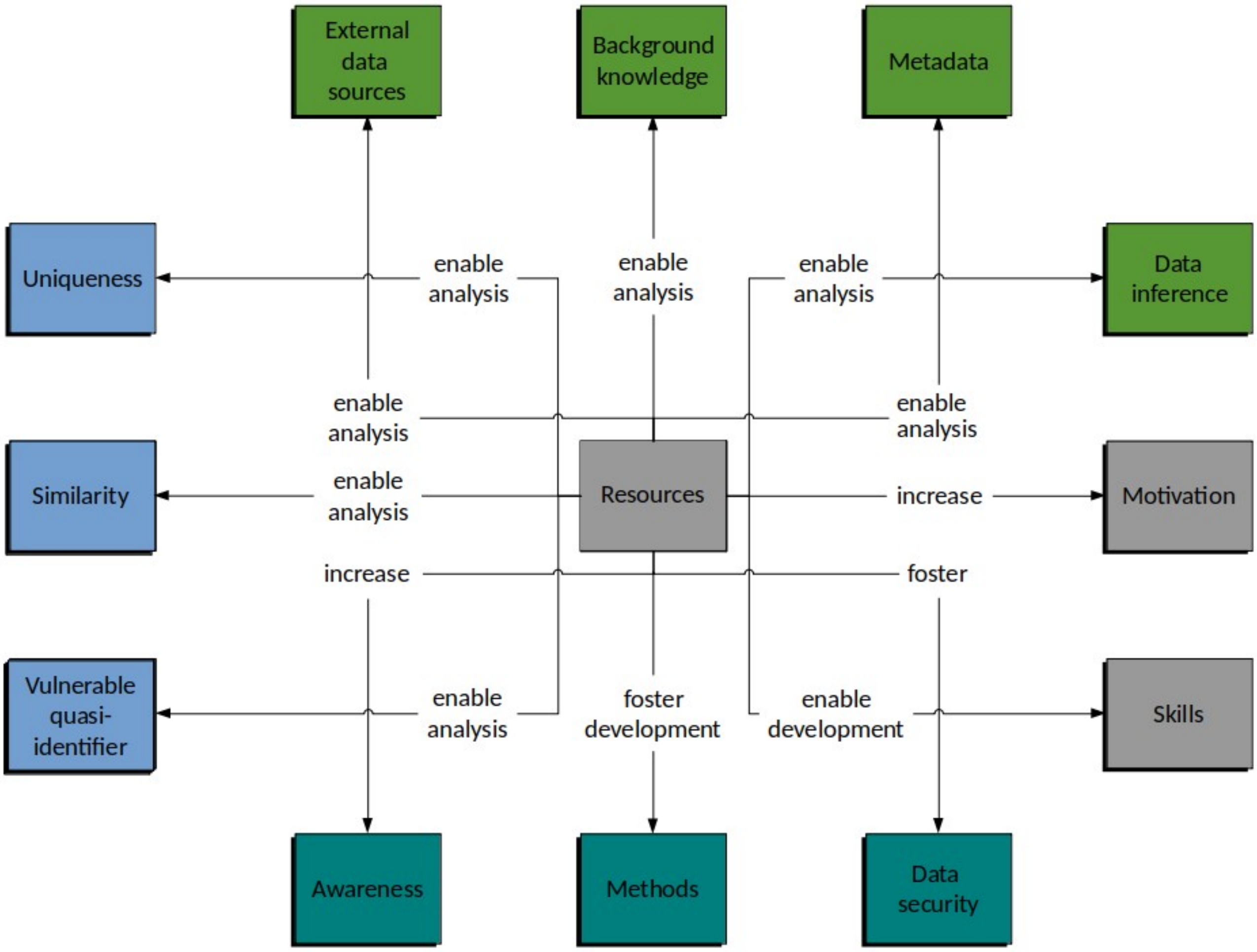

Figure 10. Interrelationships of the entity "Resources".

3.4. Interrelationships of the entities of the technical/organisational perspective

3.4.1. Awareness

The awareness of an organisation can have an indirect effect on the entities "uniqueness", "similarity" and "vulnerable quasi-identifiers" through the implementation of guidelines. For example, limit values can be defined or the use of particularly risky identifiers can be prohibited for processing purposes.

Awareness has no direct influence on the knowledge perspective, but is itself influenced by its entities. One exception is metadata, the processing of which can be regulated or prevented by a corresponding policy.

If an attacker knows that an organisation ascribes great importance to data protection, it is conceivable that this will have an inhibiting effect on their motivation. The other entities are not influenced by an organisation's awareness.

There is a strong direct effect on the entities "methods" and "data security", as a well-developed awareness drives the development and improvement of both factors.

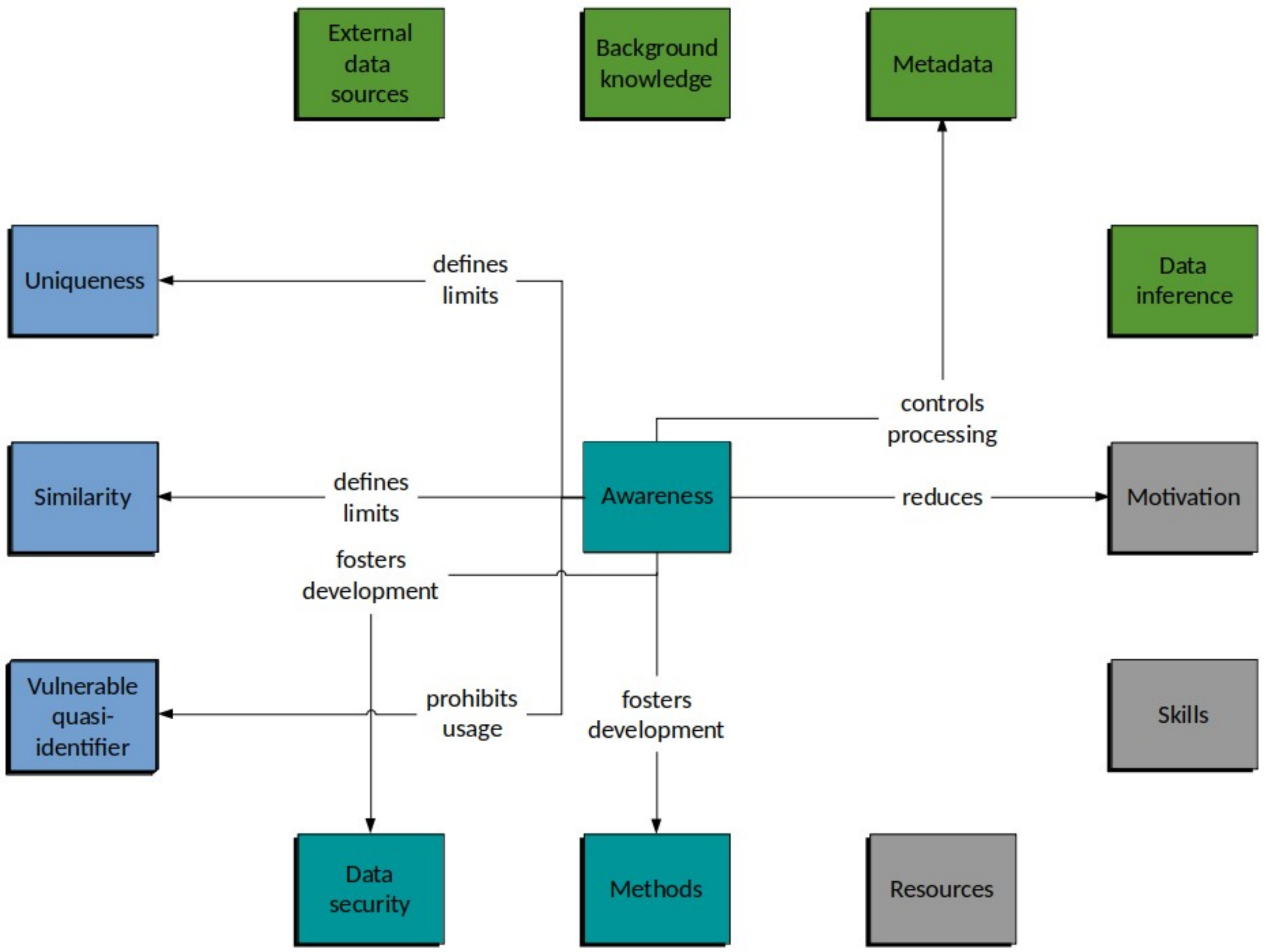

Figure 11. Interrelationships of the entity "awareness".

3.4.2. Methods

The anonymisation methods used in the provision of personal data have an active effect on the entities of the data perspective. They reduce the uniqueness and similarity of datasets and mask vulnerable quasi-identifiers. There is therefore a strong interaction.

There is no method that can remove external data sources or background knowledge from the risk assessment. Metadata, on the other hand, can be influenced by suitable methods as it is stored together with the actual user data. It is therefore conceivable to suppress metadata completely or to reduce its informative value in other ways. Depending on the type of data available, it is also possible that the methods used will affect the inference of the data. However, the respective context is decisive here.

If potent methods are used to protect patient data, this can be assumed to have both an inhibiting and a promoting ("now more than ever") influence on the motivation of an attacker. In the sense of an "arms race", it is also conceivable - as described in the literature using the example of *k*-anonymity - that a new protection method will open up a new avenue of attack. With regard to the

resources available to an attacker, it is easy to see that strong protection measures use up the available resources. It should be noted, however, that a lack of method implementation does not lead to an increase in resources.

With regard to the technical/organisational perspective, the methods used can be said to interact with the aspects of data security.

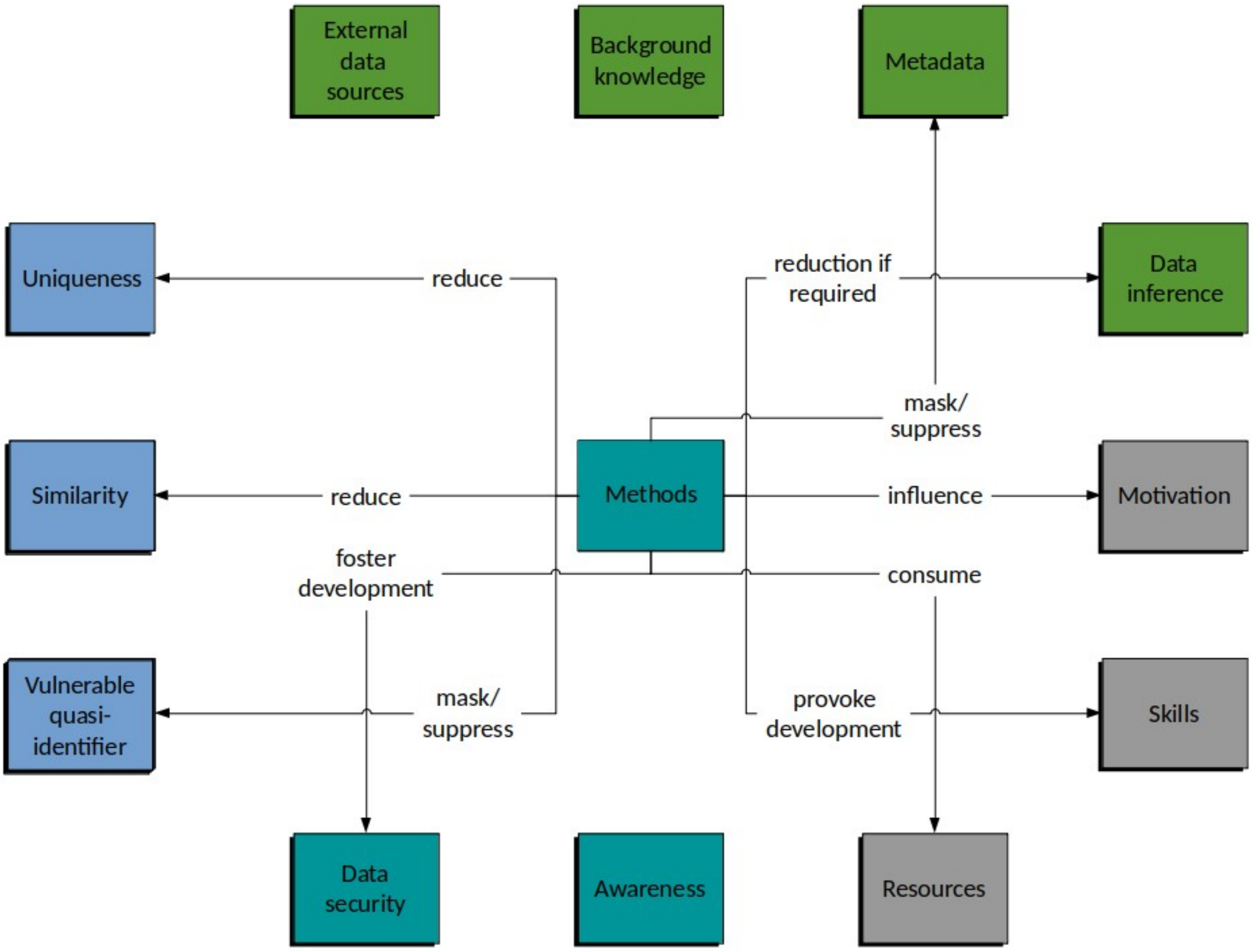

Figure 12. Interrelationships of the entity "Methods".

3.4.3. Data security

It is plausible that data security precautions fulfil a protective function vis-à-vis the entities of the data perspective by regulating confidentiality, access, etc. This is not always the case. However, the effect is considered to be relatively weak.

Analogous to the above, data security instruments have no interactions with external data sources or possible background knowledge. Metadata, on the other hand, can be suppressed or only made available to a specific group of people through strict authorisation management. Explicit technical measures against the detection of data inferences are difficult to imagine due to the almost impossible predictability.

Similar to the "methods" entity, it is conceivable that data security measures can both inhibit and encourage the drive of a potential attacker. It is also obvious that appropriate precautions protect against attacks, but also encourage the development of new approaches. It is immediately obvious that a high level of data security consumes the resources of an attacker. However, a lack of data security methods does not lead to an increase in resources.

With regard to the technical/organisational perspective, anonymisation methods and technical data protection are to be regarded as related in terms of their objectives.

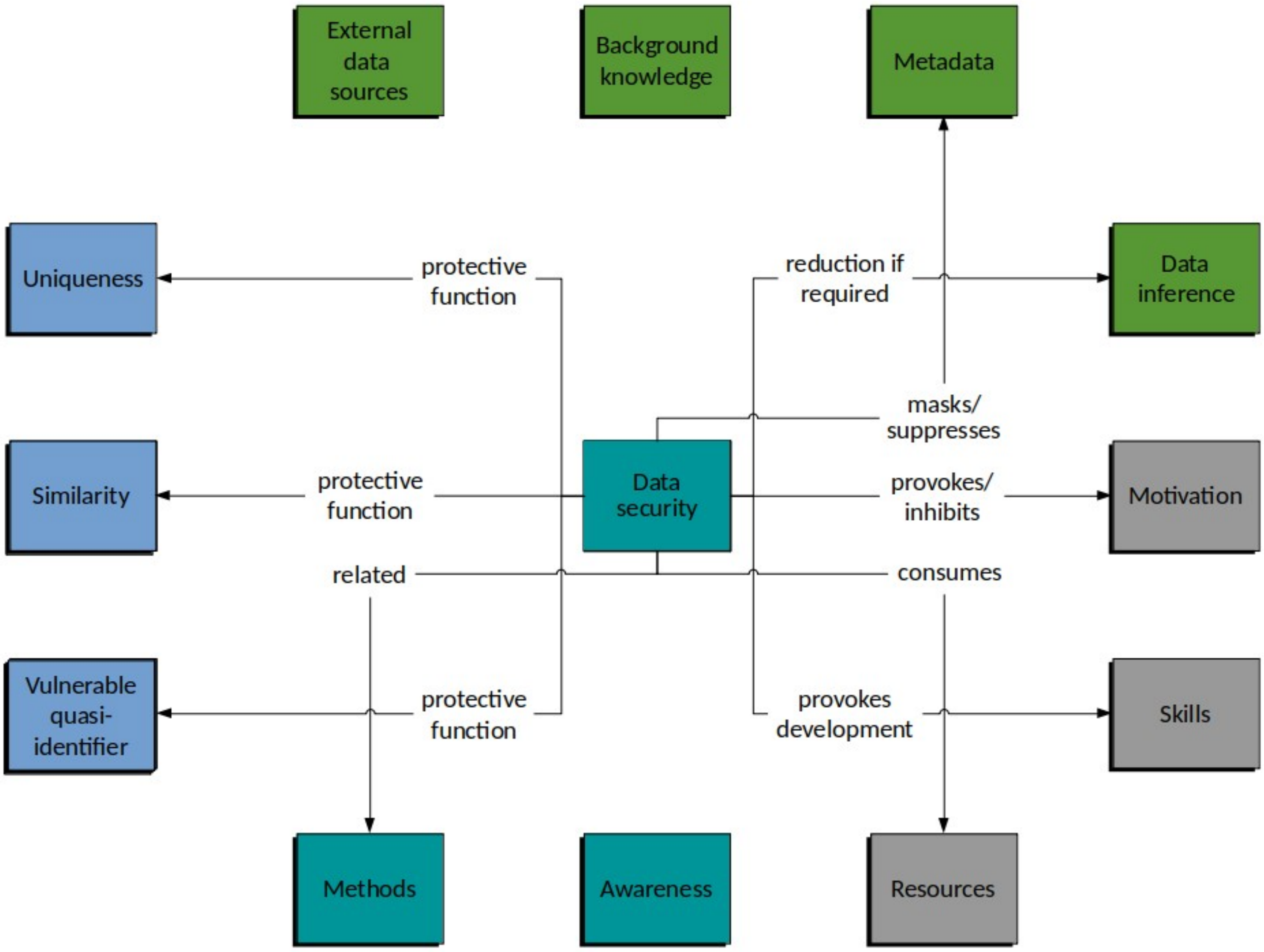

Figure 13. Interrelationships of the entity "data security".

In summary, the subject of this section was the presentation of the interrelationships that emerge between the individual risk factors. These effects were visualised in this context with the help of entity-relationship models, whereby each entity was discussed separately. The overall view revealed complex networks of relationships in which virtually every factor considered is cross-linked with the other components.

4. Influence Matrix

The representation of the interrelationships between the entities of the respective perspectives shows a clear cross-linking, which in the synopsis results in the picture of a complex system. However, ER models can only describe existing relationships qualitatively; the quantitative characteristics of the interactions between the entities cannot be represented satisfactorily in this way. Hence, in order to be able to systematically show the dynamics of these connections, a so-called influence matrix is created, based on the procedure described in the Methods section.

To present a risk assessment of a concrete dataset, it is necessary to combine the influence matrix with the weighting components of the individual entities. For this purpose, the absolute values of the interactions in the matrix are multiplied by the identified characteristics of the entities. As described earlier, the more pronounced a factor is, the stronger its effect on the "Re-Identification Risk" system, e.g. an attacker with high methodological skills has a stronger effect on the system than an attacker with low methodological skills. The resulting indices are then plotted in evaluation diagrams, where they allow the re-identification risk to be estimated or classified, for example, by means of colour coding.

Based on the ER models and related explanations, the influence matrix depicted in Figure 14 is proposed for the "re-identification risk of patient data" system. The numbers in the cells correspond to the weighting of the correlations identified in the respective ER models.

		Influence on	Data			Knowledge				Attacker			Techn. / organ.		
			D1	D2	D3	K1	K2	K3	K4	A1	A2	A3	TO1	TO2	TO3
Data	D1	Uniqueness	###	6	6	6	6	6	4	6	4	-1	2	4	4
	D2	Similarity	3	###	2	2	2	2	2	2	2	-1	1	2	2
	D3	Vulnerable QI	3	2	###	3	2	2	2	2	2	-1	1	2	2
Knowledge	K1	External data sources	3	3	3	###	3	3	3	3	2	-1	1	2	0
	K2	Background information	2	2	2	3	###	3	3	3	2	-1	1	2	0
	K3	Metadata	3	3	3	3	3	###	3	3	2	-1	1	2	2
	K4	Data inference	1	1	2	3	3	3	###	1	1	-2	1	2	1
Attacker	A1	Motivation	0	0	0	0	0	0	0	###	3	3	1	1	1
	A2	Skills	3	3	2	3	3	3	3	3	###	3	1	1	1
	A3	Resources	2	2	2	2	2	2	2	3	3	###	1	1	1
Techn. / organ.	TO1	Awareness	-2	-2	-3	0	0	-3	0	-1	0	0	###	3	3
	TO2	Methods	-6	-6	-6	0	0	-6	-4	2	4	-6	0	###	4
	TO3	Data security	-1	-1	-1	0	0	-3	-1	2	1	-3	0	2	###

Figure 14. Matrix of influence for calculation of the re-identification risk

In this matrix, the entities "uniqueness (D1)" and "methods (TO2)" are listed with double point values. These adjustments were made as part of the evaluation of the method, which showed that both entities have a disproportionately high influence on the re-identification risk or are more important than the other factors.

The majority of the attacks or assessment approaches examined targeted uniquely configured datasets as the main risk factor. Just as a fingerprint identifies a person, a unique combination of characteristics of a different kind can also serve this purpose. Determining the number of unique instances in a data collection is also concretely computable as a single entity and thus safe from subjective influence or variation. Moreover, the dominance of the uniqueness criterion can be well countered by appropriate methods. In this respect, a fundamentally stronger effect on the system under consideration can be attributed to both entities; this effect is mapped by double point values.

The entity "Resources (A3)" must be considered separately because, due to the mechanics of the influence matrix, the entities of the data perspective would lead to the effect of an ever-increasing consumption of resources by an attacker with rising degrees. From the resource perspective, however, it is irrelevant how pronounced the respective entities are. The effort required for the software-based determination of the corresponding values remains more or less the same, so a constant value of "-1" is defined here. The same is true for the knowledge perspective; here, too, an increasing proficiency or easier availability of the entities would lead to greater resource consumption. Hence, it is plausible that easier availability leads to less resource consumption because less effort is required to obtain more detailed information.

Finally, the entities "Methods (TO2)" and "Data Security (TO3)" would lead to the attacker's resource endowment improving in the case of no specification and the associated score of "-3". This effect is not conclusive; rather, such a situation leads to the fact that the attacker's resource endowment is not used up and thus there is no influence on the system.

The double point values of the uniqueness and the methods and the constant resource values are visibly integrated. The opposing effects with regard to the resource endowment were implemented by means of (nested) If-constructs in the differently coloured cells in the spreadsheet (see supplemental materials).

4.1. Risk assessment

The assessment of the re-identification risk of a specific dataset is carried out in two steps:

1. In order to initiate the automated calculation of the re-identification risk of a specific dataset in the influence matrix, the data must be assessed by the analyst, data controller, or any other responsible person with regard to the weighting of the entities of the risk perspectives.

   Figure 15 shows the input template for the scores of the individual factors.

Determining the weighting of the entities:
The entities under consideration build the system which is formally represented in the influence matrix. They have different effects on the system, depending on their weighting. The weighting must be entered in this template.

The following applies:
0 points corresponds to a non-pronounced entity
1 point corresponds to a weakly pronounced entity
2 points correspond to a moderately strong entity
3 points correspond to a strongly pronounced entity
-3 points are awarded as a “penalty” if the entities of the technical/organizational perspective are not pronounced

			Score
Data	D1	Uniqueness	1
	D2	Similarity	0
	D3	Vulnerable quasi-identifier	1
Knowledge	W1	External data sources	3
	W2	Background knowl./ context	2
	W3	Meta data	0
	W4	Data inference	1
Attacker	A1	Motivation	3
	A2	Skills	3
	A3	Resources	2
Techn. / organ.	TO1	Awareness	3
	TO2	Methods	3
	TO3	Data security	2

Figure 15. Spreadsheet template for entering weighting scores

2. The absolute weighting values determined are then multiplied by the absolute values of the effect relationships . The indices of the impact matrix change and show the magnitude of the re-identification risk.

Finally, a graphical representation visualises the magnitude of the risk. An Excel spreadsheet is available for the practical implementation.

Based on the hypothetical characteristics of the influencing factors, a "risk range" is defined with corresponding upper and lower limits within which the probability of an undesired re-identification of a dataset is to be classified. The range of values between the maximum and minimum expression of the influencing factors is split into evenly subdivided sections, representing a gradual increase or decrease of the re-identification risk.

The total risk of an undesired re-identification that can be assessed here thus covers a range of values between 28 and 1670 points, and the risk range is correspondingly 1642 points. In order to be able to make a statement about the degree of risk, the range is divided into equally sized categories:

- 0 - 28 points: "base risk", low risk of re-identification that cannot be influenced any further, cannot be ruled out even with best possible risk management
- 33 - 579 points: moderately high risk; re-identification of medical data is conceivable in principle
- 580 - 1125 points: high risk; successful re-identification is probable
- > 1125 points: very high risk; successful re-identification can be assumed

The visualisation of the Q-values which provide measures of the cross-linking of the influencing factors can also be done as a bar chart. Positive Q-values indicate a risk-increasing, negative Q-

values a risk-reducing effect of a factor under consideration. The evaluation diagram of the Q-values provides useful indications of ways in which the re-identification risk of a particular dataset can be effectively reduced. From the diagram in Figure 16, it can be seen that with maximum detectable risk level, dataset uniqueness (D1) and metadata (W3) are very strong risk drivers and should be addressed accordingly. In contrast, the quality of methods (TO2) exerts an inhibiting influence on the system for minimum attribute values and is thus determinant for the total risk expression with minimum values.

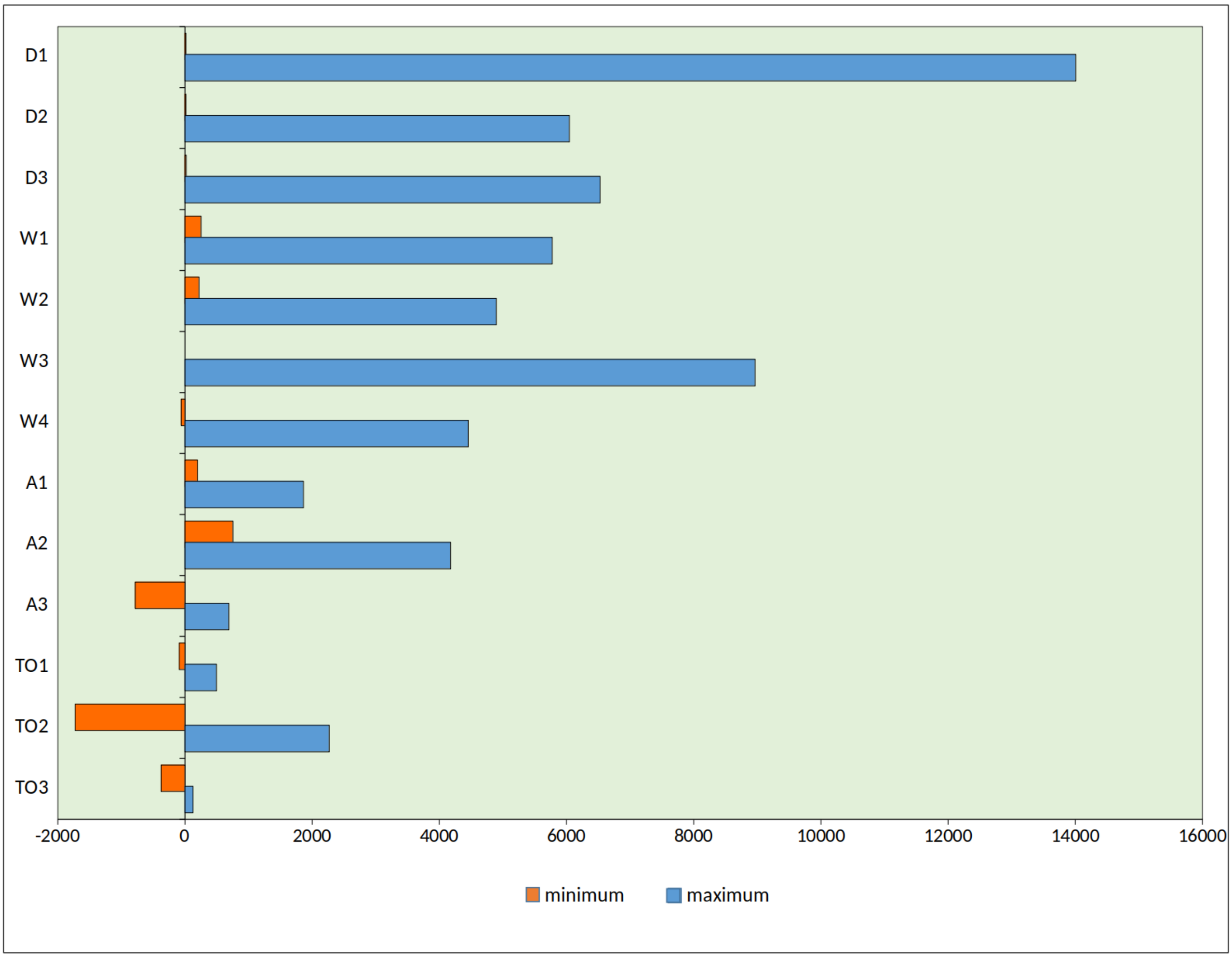

Figure 16. Graphical representation of the maximum and minimum Q-values of the influencing factors.

The active sums of the influencing factors cover a range from -72 to 161 points, the passive sums are in an interval between -34 and 108 points. The calculated active and passive sums of an influence factor are plotted as a pair of values in a Cartesian coordinate system; the active sum is plotted on the abscissa and the passive sum on the ordinate. For better representability, the value ranges of the sums are rounded to the next larger representable numerical value. The diagram is to be provided with subdivisions of equal size, which represent the increasingly strong expression of the value pairs (and thus also of the total risk).

The following information can be generated from the evaluation diagram:

1. The total risk correlates directly with the expression of the influencing factors. Hence, the diagram is suitable for estimating it, but is not as intuitive as the visualisation of the risk score.
2. The strength of this representation lies in the possibility of being able to analyse the position of a certain factor in the system. The extent to which a factor actively influences the risk or how much it is influenced by the other components can be estimated. For this purpose, the categories of influencing factors presented above can be used. In this respect, the presentation therefore complements the visualisation and usefulness of the Q-values.

Figure 17a and 17b show the position of the individual pairs in the coordinate system under minimal or maximum expression of the risk factors

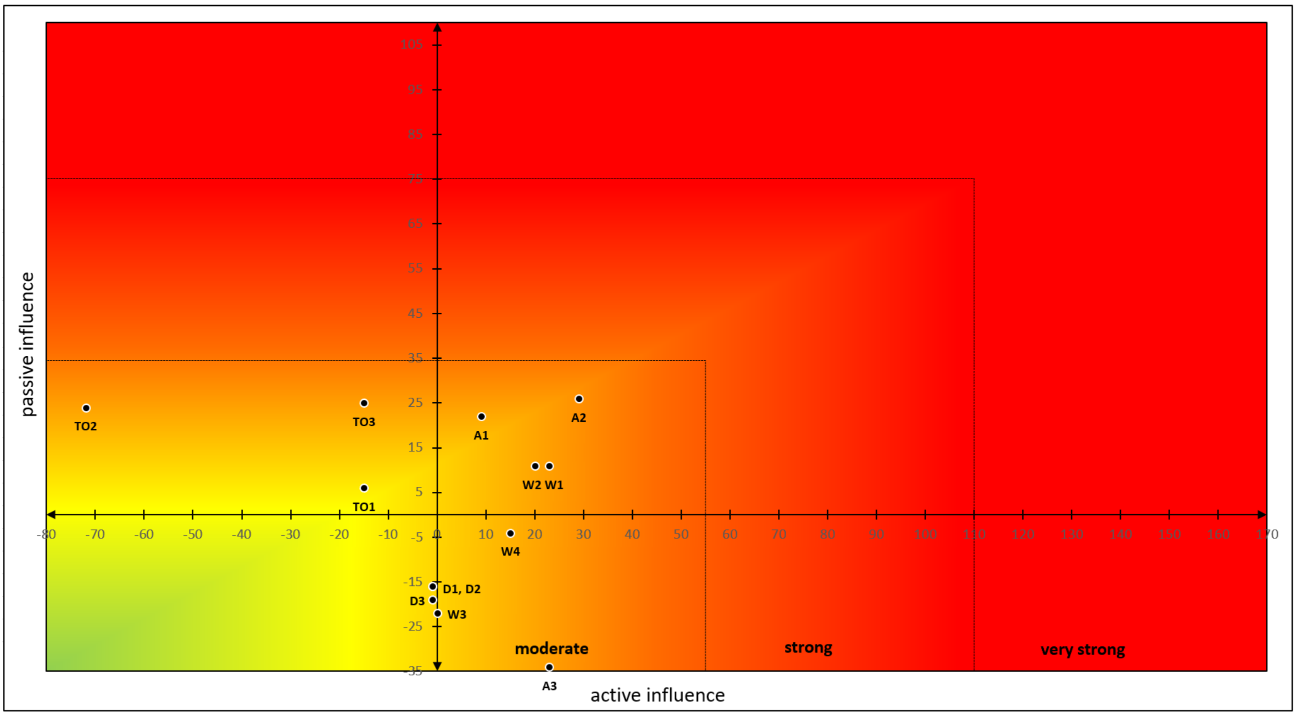

Figure 17a. Differentiation of the smallest Re-ID risk according to influencing factors.

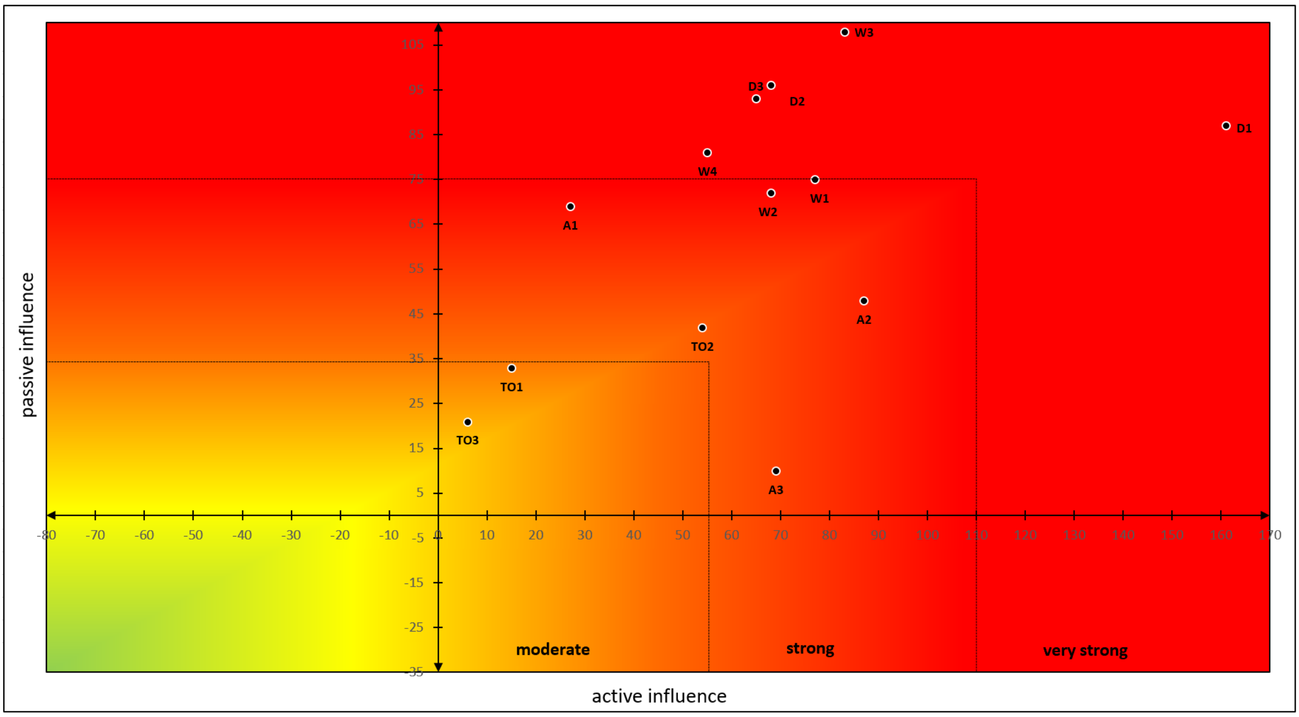

Figure 17b. Differentiation of the highest Re-ID risk according to influencing factors.

Use of the results and recommendations for action

Explicit recommendations for action can be derived from the results of the risk assessment. However, the usefulness or informative value of these recommendations depends on which of the indices discussed is used as the basis for such a proposal.

First of all, the overall risk value or risk score is suitable as a relatively unspecific indication for primary risk assessment. In the sense of a "traffic light", it provides an indication of whether the data collection under consideration poses an acute risk of unwanted re-identification. However, it does not provide a differentiated and, above all, transparent presentation of the risk components, which is why no specific recommendations for action can be derived.

The Q-values determined and the differentiation of the overall risk into its influencing factors are much better suited to the possible management of the re-identification risk. This provides both a categorisation of the overall risk and a breakdown of this risk potential into its factors.

The benefit of the Q-value as a measure of cross-linking for risk management is demonstrated by the possibility of exerting a targeted influence on the extent of the re-identification risk with a high Q-value in combination with a controllable or customisable influencing factor. In this context, it should be noted that the factors can either increase or decrease the probability of occurrence of this risk, as shown in Table 5.

Table 5. Overview of influencing factors and their direction of impact.

Controllable influencing factors ("risk levers")	Effect	Non-controllable influencing factors	Effect
Uniqueness	increasing	External data sources	increasing
Similarity	increasing	Background knowledge, context	increasing
Vulnerable quasi-identifier	increasing	Motivation	increasing
Metadata	increasing	Skills	increasing
Data inference	increasing	Resources	increasing
Awareness	lowering		
Methods	lowering		
Data security	lowering		

In order to reduce the re-identification risk of a data collection, factors with risk-increasing potential must be weakened in their effect on the system and thus their Q-values reduced. At the same time, factors with risk-reducing properties should be strengthened in their effect and their Q-values increased. Depending on the entity in question, different measures are available for this purpose, some of which is referred to in the Definitions and Concepts section above.

As an illustrative example, let the Q-value of the metadata in a situation under consideration be strongly positive. In this case, the metadata can be suppressed with appropriate technical and organisational measures, which leads to a reduction in the re-identification risk. If, on the other hand, ceteris paribus, the Q-value of the attacker's skills is strongly positive, there are no control instruments available in this case, as this factor cannot be influenced externally.

Reduction of uniqueness

In order to reduce the number of unique data records within the data collection, generalisation approaches are primarily suitable, whereby data is combined into hierarchically organised categories with a common characteristic and a "coarsening" and thus a dissolution of the uniqueness of the data records is achieved.

Example: Blood values are requested from prostate cancer patients; the PSA value should be as specific as possible, while all other parameters may be subject to a loss of information. Accordingly, these characteristics are generalised, but the PSA value relevant for further analyses remains untouched.

Increasing the similarity

If datasets are secured against an excess of unique characteristics with the help of generalisation methods, more similar datasets are generated at the same time. As a result, the risk potential of this feature is reduced. In principle, the "noise addition" of data attributes is also suitable because it can be used to break up the unique configuration of data records and create a certain similarity by adding "fuzziness".

Dealing with vulnerable quasi-identifiers

Since there is a strong interaction between the entity "uniqueness" and the "vulnerable quasi-identifiers", the same methods already discussed in connection with the reduction of uniqueness are basically suitable for reducing the risk potential of these characteristics. Here too, the possible loss of information after appropriate processing must be taken into account.

Ideally, the vulnerable quasi-identifiers are simply deleted from the data collection under consideration, but this procedure is not suitable for diagnostic codes because it would remove a central information carrier from the data records. In this respect, generalisation methods are also the method of choice here in order to achieve a certain protective effect.

Handling of metadata

Metadata describes information about the actual data, such as its source, type of creation, structure, etc. and is important for the reuse of medical data, especially if it contains information about the

data quality [58,69]. However, the majority of metadata is not required for analyses and should be removed before the data records are released or at least coarsened to such an extent that it does not pose an increased risk. The methods for doing this depend on the metadata in question.

Dealing with data inference

There is no generally applicable method for determining the dimensions of a possible data inference. In principle, however, it can be assumed that the more "fuzzy" the datasets become, the less likely it is to derive correlating information from the available data. Generalisation and randomisation measures can therefore be suitable for reducing the potential for data inferences. In addition, the suppression of datasets that are assessed as particularly susceptible is available as a last resort.

The problem of information loss

All of the methods described so far are accompanied by a loss of information or reduced informative value of the datasets. In extreme cases, this loss of information can make further evaluation of the data impossible and lead to so-called "over-scrubbing" [70]. Although it is possible to estimate the resulting loss quantitatively using the metrics presented in the Definitions and Concepts section above, domain knowledge and therefore the expertise of a specialist is required for an evaluation.

If the patient data must not be altered in any way because the associated loss of information is unacceptable, pseudonymisation of the data records is an option. The use of hashing algorithms is suitable for this. Anatomy and slicing are other methods that leave the information content of the data records untouched. Alternatively, it is also possible to completely separate identifying features from the sensitive attributes and store them with a data trustee [71].

It should be noted, however, that when using these methods, data-inherent properties such as a unique dataset configuration and the resulting re-identification risk are not are not dealt with.

Creating awareness

Awareness should actually be a matter of course when dealing with medical data. However, if it is discovered that the executive and therefore responsible level of the organisation is not aware of the problem of the re-identification risk of patient data, the challenge is to raise this awareness. The best way to do this is to outline the legal consequences for the organisation and at the same time present a package of measures to reduce the risk of re-identification.

Quality of methods

In order to effectively reduce the risk of re-identification, methods such as a generalisation or suppression approach must be applied to the data collection. The quality of the methods or the degree of anonymity achieved can then be determined using the formal anonymity criteria as presented in the Definitions and Concepts section above.

It is advisable to apply a combination of methods to the data collection that satisfies an anonymity criterion that has not yet been successfully attacked. For example, only a generalisation approach can be chosen whose quality can be determined with the help of *k*-anonymity. Meanwhile, the literature already provides descriptions of successful attacks (in the sense of reconstructed personal references) on correspondingly treated data collections, so that the concept of *k*-anonymity and thus also the exclusive use of this method for generalizing the data must be regarded as broken [47]. But the privacy requirement of *k*-anonymity should only be a starting point for privacy protection, and further steps should follow to prevent the disclosure of attributes that may otherwise be possible due to value homogeneity and external knowledge attacks on [72]. For the handling of patient data, it is therefore recommended from a data protection perspective to use a bundle of methods that at least meets the criteria of t-closeness or differential privacy. However, this recommendation does not apply without restriction, especially as data utility must again be kept in mind. In addition, restrictions regarding existing workflows must be expected, especially when using differential privacy, so that a thorough consideration must be made between data protection and the usability or purpose of the data [73].

Data security

In order to achieve an acceptable level of data security, certification options are available. However, the preparatory work required for this is not insignificant in terms of personnel, technical and financial resources. Depending on the resources of the organisation in question, it may therefore make sense to focus solely on the standards (usually ISO 27001) without aiming for final certification.

Dealing with potential attackers

Although the entities of the attacker perspective have been defined as non-controllable, the possibility of contractual and legal regulations should nevertheless be pointed out here in addition.

The recipients of medical data and therefore potential attackers are usually researchers and, for example, university staff. With the help of procedural instructions or so-called Data Use Agreements (DUAs), data users can be obliged, for example under threat of sanctions, not to make any attempts at de-anonymisation and to handle the patient data they receive responsibly (e.g. no

sending of sensitive data by email or the mandatory use of a cryptographic solution for mobile devices). Although the skills and resources of an attacker cannot be influenced in this way, it is conceivable that the incentive to carry out an attack could be reduced. However, all contractual efforts are invalidated if the data falls into unauthorised hands, for example due to technical circumstances.

In summary, the indices that can be derived from the influence matrix provide indications of particularly risk-driving components of a medical data collection. These clues can then be treated accordingly in order to counter the risk of de-anonymisation. In this context, generalisation, suppression and randomisation approaches play a decisive role. In principle, however, the application of these methods results in a more or less pronounced loss of information, which reduces the suitability of the data for secondary use. Consequently, the attempt to prevent a possible reconstruction of a personal reference is an optimisation problem: on the one hand, the highest possible level of security should be achieved, while on the other hand, the associated loss of information should be as low as possible.

The following section provides an evaluation of the procedure based on predefined scientific quality criteria by means of retrospective application tests in two published successful re-identification attacks.

Evaluating the procedure and determining its method quality

The method presented here is based on a heuristic approach, which, with limited information and the intervening effect relationships, attempts to provide an indication of the probability of re-identification of patient data. Heuristics are by definition prone to error. Therefore, an experimental application is attempted to obtain a statement about the goodness of this method or to gain an impression of whether the heuristic can provide useful results with regard to a risk assessment. In this context, criteria are first defined which are to be used for the evaluation of the heuristic.

The method is then used to retrospectively analyse the risk potential of some of the re-identification attacks described in the scientific literature (or rather the data considered in these attacks). Thereby, the perspective of a data protection officer is taken, who has to assess the thematised datasets before releasing them for secondary use. Finally, the results obtained and the procedure itself are assessed on the basis of the quality criteria.

The retrospective view has the advantage that there is corresponding evidence of a successful re-identification, which serves as an important indicator for the assessment of method quality. A disadvantage is that often not all entities of the risk perspectives are presented in the publications, and thus the re-identification risk presented in the publications and, accordingly, the re-identification risk is not fully captured.

In the literature there are no generally valid criteria to assess the quality of a heuristic. Therefore, the evaluation of the presented method will be based on the test quality criteria known from the scientific literature [74].

a) *Solution quality:* As the most important criterion for the evaluation of a heuristic, the comparison between the result of the applied heuristic and an optimal solution is regularly proposed, if this can be determined. In this context, the term "solution quality" is used [75]; this criterion can be compared with the concept of "validity" in the context of test quality criteria.

   No optimal solution is available for the risk assessment considered here. The quality of the solution is to be determined by checking whether the result of the risk estimation and the result of the re-identification attempt form a plausible relationship. If the heuristic indicates a low risk potential, while the executed attack, on the other hand, describes several secured recovered person references, a low solution quality is to be assumed. Conversely, the solution quality is high if a high probability of occurrence was detected.

b) *Plausibility*: The presentation of the Q-values as well as the factor differentiation reveal entities that have a particular influence on the re-identification risk. Accordingly, the factors

thus identified can be used to reduce the risk, provided that a possibility to adjust these items exists.

The plausibility or comprehensibility of these indications is to be assessed as an evaluation criterion. This means that the indications of particularly influential factors in the overall analysis should be coherent and not logically mutually exclusive. This would be the case, for example, if there were high values for vulnerable quasi-identifiers, but the uniqueness of the datasets was low.

c) *Discriminatory power:* This criterion is intended to show whether risk assessments differ sufficiently from one another under changed conditions; in the context of test quality criteria, this is referred to as "change sensitivity". This aspect is important, for example, if after an initial risk assessment measures have been taken to safeguard the data and a new run of the heuristic is carried out for verification. It should now be possible to determine a clear difference in the risk assessment.

d) *Effort*: The effort criterion is intended to assess the economy of methods, with the key question being: What resources must be deployed in order to be able to make a statement about the risk potential of a particular data collection? Analogous to the entity "attacker's resources," this is intended to estimate what time, technical and expert resources are required to assess the risk.

Application and evaluation

First application test

In a much-cited paper from 2002, Latanya Sweeney demonstrates a linkage attack on a database with medical data from over 135,000 people and their families [76]. Around 100 data attributes were available for each person, including information on their zip code, date of birth and gender. This data collection was considered "anonymous" and made available to science and research for further use. As an additional external data source, Sweeney bought a freely available voter register for $20, which contained the names of the voters, their address, zip code, date of birth and gender. By linking the zip code, date of birth and gender information from both data sources, Sweeney was able to locate the medical record of the state governor at the time.

For a retrospective assessment of the re-identification risk, the entities of the risk perspectives are to be considered and weighted using the information from Sweeney's publication [76].

- *Uniqueness*: Sweeney was able to show that 87% of the US population is uniquely identifiable in terms of zip code, date of birth and gender [77]. In combination with around 100 other data attributes, it can be assumed that the datasets are very unique. 3 points.
- *Similarity*: Presumed occurrence of similar data records based on the quantity of data records and recorded data attributes; however, no information is provided regarding this entity. To take the existence of similar data records into account, at least to some extent, 1 point is awarded.
- *Vulnerable quasi-identifiers*: No clear indications as to which vulnerable quasi-identifiers are present. The characteristics "zip code" and "diagnoses" are definitely mentioned; in addition, it is very conclusive that the data records also contain the other quasi-identifiers. 3 points.
- *External data sources*: Sweeney was able to acquire a collection of data suitable for identification with little effort. This fact justifies the award of 3 points.
- *Background information:* This entity was not described in the publication; however, the complete lack of background information is unrealistic. Therefore, the lowest possible point value of "1" is given.
- *Metadata:* This entity was also not described in the publication; it is assumed here that no metadata is available. Accordingly, zero points are given.
- *Data inference:* This entity was also not described. Due to the amount of available patient information, however, a pronounced inference potential is plausible, so that 3 points appear justified.
- *Motivation of the attacker:* For Sweeney, as a scientist and researcher in the field of data science and data protection, the motivation to point out vulnerabilities in this regard must be considered high. The mere fact that the data records in question were available for scientific and private sector use without further examination means that a worst-case scenario must be assumed. 3 points.
- *Skills of the attacker*: As a scientist, Sweeney is methodically very well trained. Furthermore, the above reasoning can be followed if one does not want to focus on her person. 3 points.
- *Attacker's resources*: As a researcher, Sweeney has certain financial and technical resources, but not unlimited ones. Once again, however, the derivations already discussed can be followed, so that a good level of resources should be assumed. 3 points.
- *Awareness*: The alleged anonymisation of the data collection indicates at least a minimum level of awareness of data protection issues. The provision of data without further

examination of its use indicates incompletely developed guidelines. Further information on this is missing, so 1 point is awarded.

- *Methods*: Based on the information provided, it can be assumed that the attempt at anonymisation was limited to the removal of names and a few other identifiers. Further methods are not mentioned. 1 point.
- *Data security*: Although no information is provided about this entity, it can at least be assumed that a state organisation such as the one in this case has a self-developed action plan. 1 point.

The weighting of the entities in conjunction with the influence matrix results in a risk score of 1,102 points or 65.9% of the maximum re-identification risk that can be represented. The probability that a personal reference can be reconstructed from the available data records is therefore assessed as "high". Figure 18a-c show the graphical representations of the single influencing factors, the re-identification risk score and the Q-values as indices of the first application test.

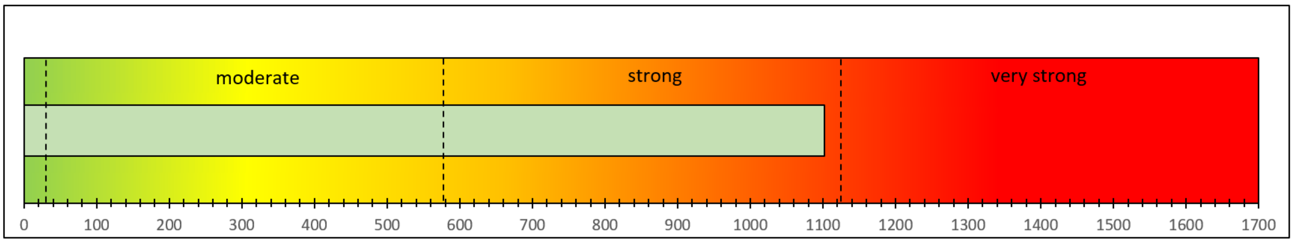

Figure 18a. Re-identification risk score.

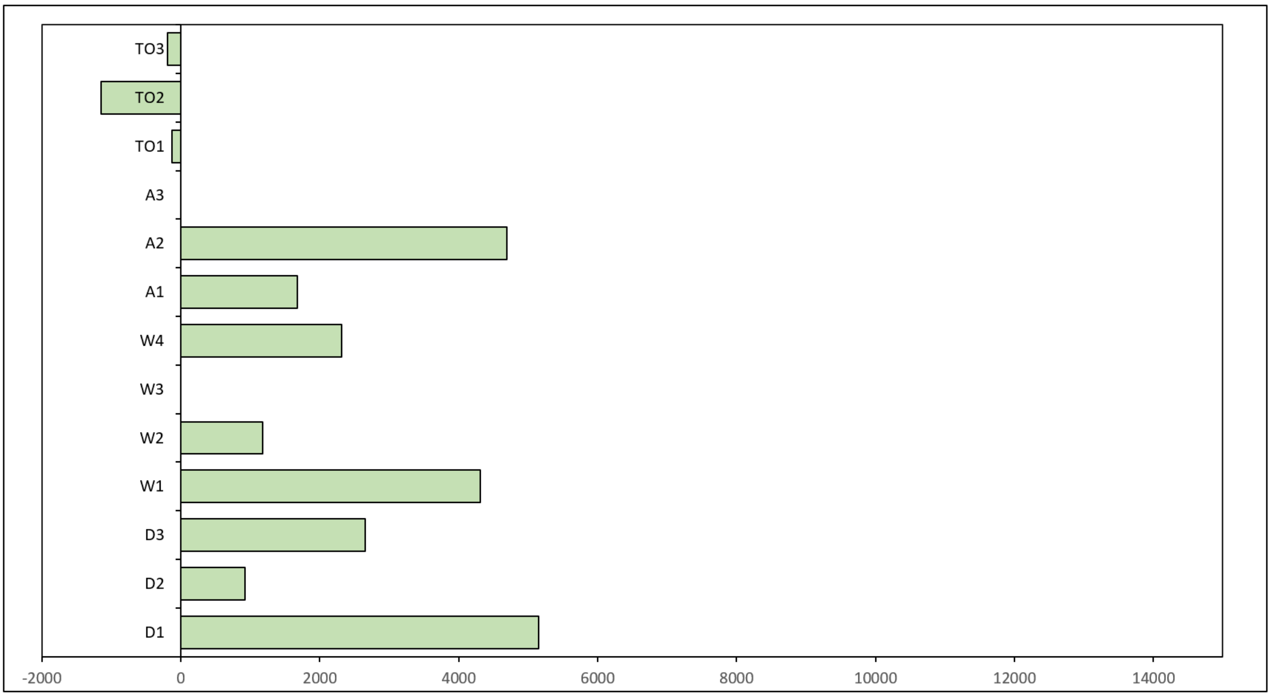

Figure 18b. Q-values of the influencing factors.

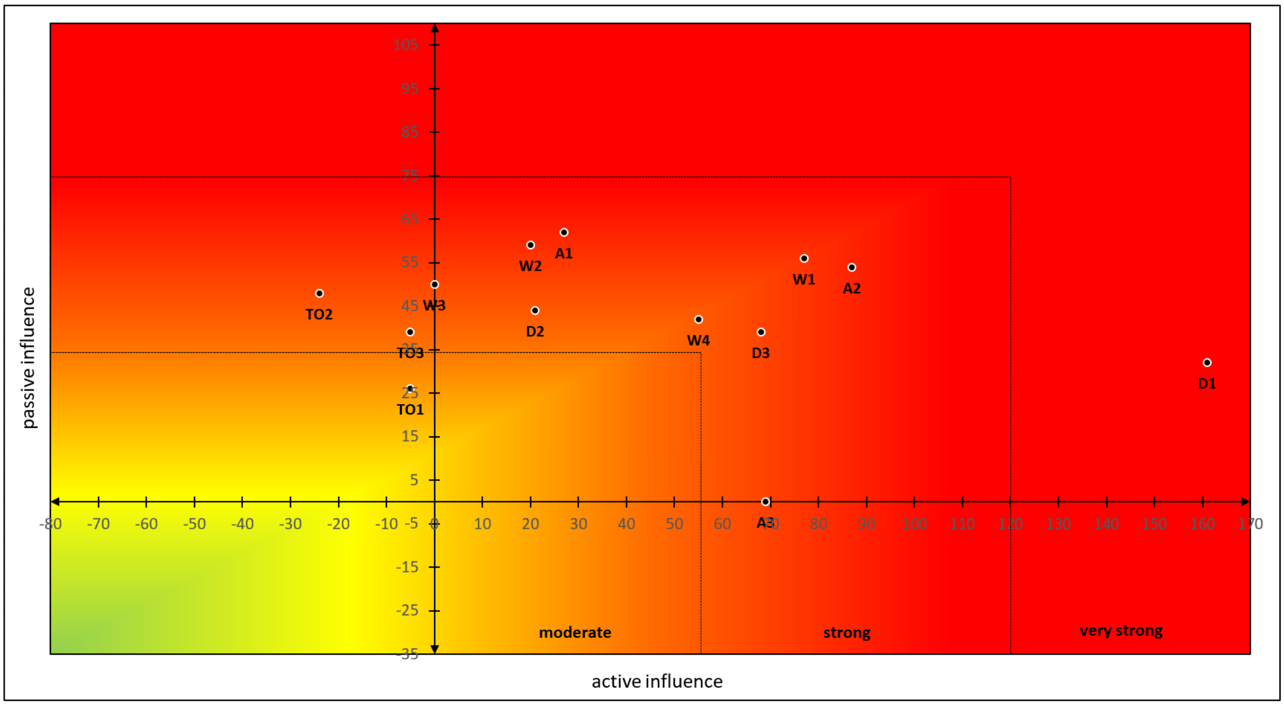

Figure 18c. Differentiation of the Re-Identification risk according to influencing factors.

With regard to the quality criteria, the following ratings result.

Solution quality: a score of 1.102 indicates a high risk of possible re-identification. This assessment is therefore basically plausible in view of the fact that the personal reference of a dataset could actually be reconstructed, but is also classified as too low.

The underestimation of the re-identification risk is probably due to incomplete information regarding the 13 interacting factors, some of which were not explicitly described and therefore had to be estimated. However, the distance to the next risk category is rather small.

Plausibility: An examination of the Q-values and factor differentiation shows that, in addition to the "skills of the attacker" (A2) and the easily accessible external data material (W1), it is primarily the pronounced uniqueness of the datasets (D1) that represents the strongest risk driver. However, the quality of methods (TO2) has the strongest inhibiting influence on the system.

This result is logically consistent with the re-identification risk model, which considers not only uniquely configured data records as a fundamental problem with regard to data protection, but also the quality of the protection approaches used as a central aspect in risk assessment. Overall, the results found are considered plausible.

Interestingly, the Q-value of the entity "attacker's resources" (A3) drops to zero in the situation described here, meaning that no cross-linking can be detected. However, a look at the factor differentiation shows that this entity has a strong active influence on the system, but is not further influenced by the other entities in the final result. This situation is not relevant for a later

recommendation for action, as the entity "attacker's resources" is considered uncontrollable anyway, and therefore cannot be influenced with regard to a possible risk reduction.

Discriminatory power: If we assume in this application example that the data records were treated with anonymisation methods after the initial risk assessment, a significant reduction in the overall risk can be observed. If the Q-values are used as a guide, and methods are applied which, as a result

- reduce the number of uniquely configured data records to less than 5% of the total data records (point value 3 → 1),
- increase the number of similar data records due to the first measure (point value 1 → 0),
- limit the number of vulnerable quasi-identifiers to one instance (such as the diagnostic codes; point value 3 → 1),
- satisfy at least l-diversity (quality of methods; score 1 → 2),
- reduce the data inference due to the effects already described (point value 3 → 1),

a risk score of 622 points is obtained, if the other influencing factors are retained. This shows a clear difference in the risk score before and after treatment of the datasets.

Effort: Overall, only a few resources are required to apply the RIMEDA procedure:

In order to assess the entities of the data perspective, the analysed datasets should already be available in a machine-readable format. Accordingly, it is possible to obtain a statement about the characteristics of these features quickly and (apart from possible license costs) without financial expense using suitable software. However, methodological knowledge is essential, so that an appropriately trained person must carry out the assessment.

With the exception of metadata, the characteristics of the entities of the knowledge perspective can only be estimated, as not only is the range of possible data sources very wide, but also the potential for data inference cannot be determined using any specific method. Overall, the material resources required for this perspective are estimated to be low, but domain knowledge is necessary to assess data inference.

The entities of the technical/organisational perspective can be assessed simply by asking questions. Although no statements are made in this regard in the present study, an estimate was made. In principle, however, the effort required for the assessment is estimated to be low; but once again, domain knowledge is required.

Second application test

In their 2021 publication, Carvalho et al. demonstrate a linkage attack on patient data from the Covid-19 pandemic in Portugal [78]. This data is collected by the Portuguese Ministry of Health and made available for secondary scientific use after the removal of identifying characteristics (in accordance with current EU legislation). At the time of the study, the data collection included 20,293 individuals, described with 15 attributes each.

For the further procedure, both the complete data collection and the subset of people who died of Covid-19 (n=502) were considered, whereby only the latter was used for a linking attempt. By linking the death data with newspaper information, a total of 12 complete personal references could be reconstructed from 192 clearly configured data records identified.

For the retrospective assessment of the re-identification risk, the entities of the risk perspectives are considered again and weighted based on the information from the publication.

- *Uniqueness*: The determined rate of uniquely configured data records can be classified as very high, both with regard to the entire data collection at 24.34% and with a focus on deaths at 38.24%. 3 points.
- *Similarity*: The authors do not provide any information on the occurrence of similar data records, but the high rate of unambiguous data records leaves little room for this. This assessment is supported by the later recording of deaths with a proportion of 99.65% clearly configured data records. 3 points.
- *Vulnerable quasi-identifiers*: Thanks to the specified data attributes, it can be seen that a total of three vulnerable quasi-identifiers are included. The diagnosis can be determined from the context of the data collection, the length of stay from the hospitalisation date. 3 points.
- *External data sources:* For the re-identification of deaths, the use of obituaries published in newspapers was obvious. 3 points due to the easy availability and good suitability.
- *Background knowledge*: This entity was not described in the publication; however, the complete lack of background information is unrealistic. Hence the lowest possible value of 1 point.
- *Metadata:* Not mentioned in the publication; therefore it is assumed that no metadata is available. 0 points.

- *Data inference*: Not described in the context of the paper. However, due to the amount of patient information available, a pronounced inference potential is plausible (and is also demonstrated in the context of the vulnerable quasi-identifiers). 3 points.
- *Motivation of the attacker*: The authors of the study are researchers at a university specializing in data science. Their motivation to develop a publishable re-identification attack can therefore be considered high. 3 points.
- *Skills of the attacker*: As in the previous entity, methodically well-trained persons can be assumed here. 3 points.
- *Attacker's resources*: The data is used in the research context, so it is assumed that there is at least sufficient availability of financial, technical and time resources. 2 points.
- *Awareness*: The medical data is collected by the Portuguese Ministry of Health and prepared for further use. The Ministry is obliged to comply with European directives on data protection, so it can be assumed that there is a high level of awareness of the need for data protection measures. 3 points.
- *Methods*: According to the publication, direct identifiers were removed from the datasets to ensure the privacy of the data subjects. Further measures were apparently not taken. 1 point.
- Data security: There is no information on this entity; however, a government organisation such as a ministry can at least be assumed to follow established data security standards. 2 points.

The weighting of the entities in conjunction with the influence matrix results in a risk score of 1,114 points or 66.7% of the maximum re-identification risk that can be represented. The probability that a personal reference can be reconstructed from the available data records is therefore assessed as "very high". These results are visualised in the evaluation diagrams in Figure 19a-c.

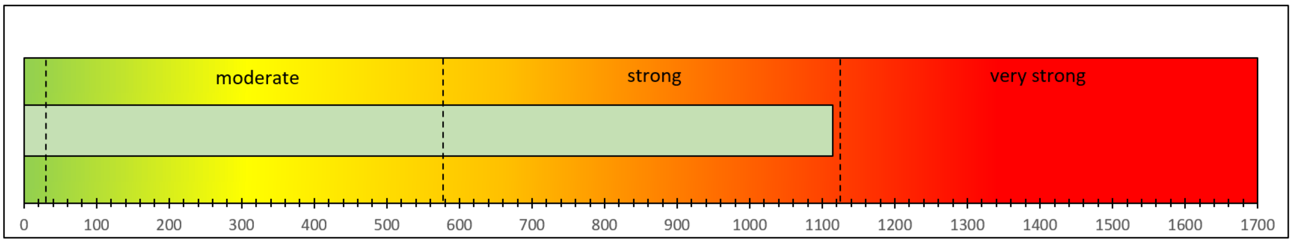

Figure 19a. Re-identification risk score

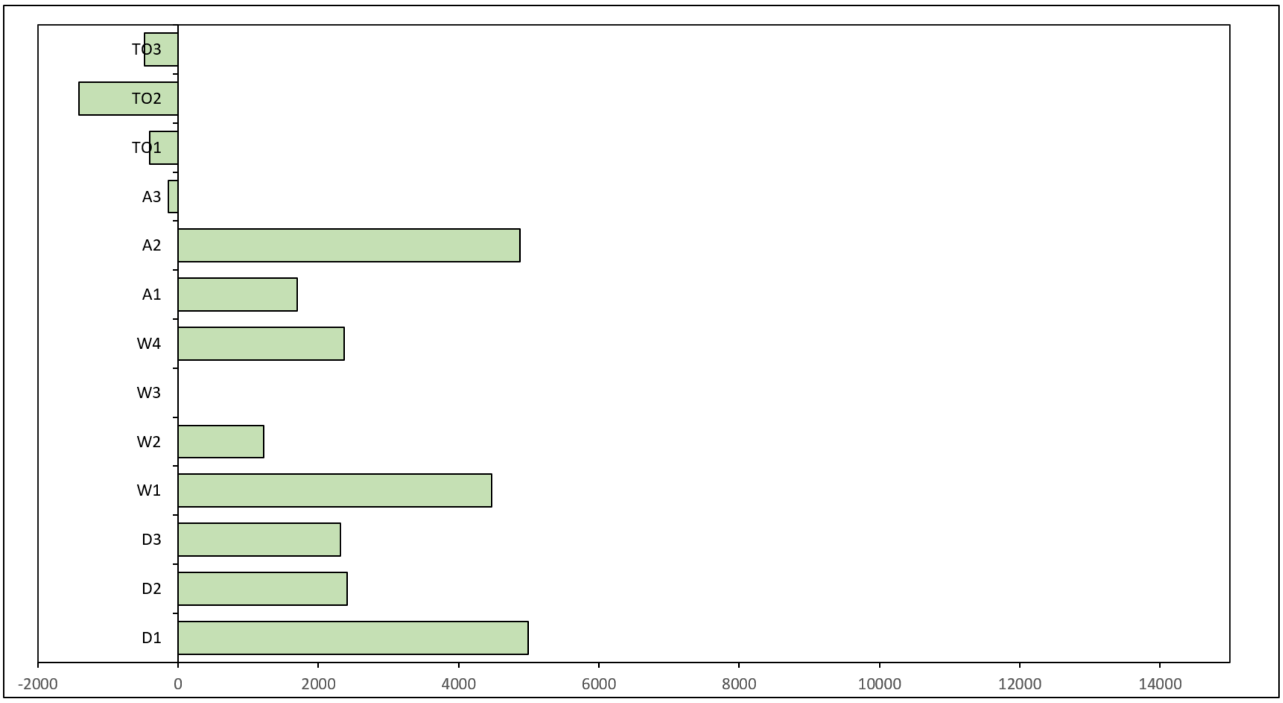

Figure 19b. Q-values of the influencing factors.

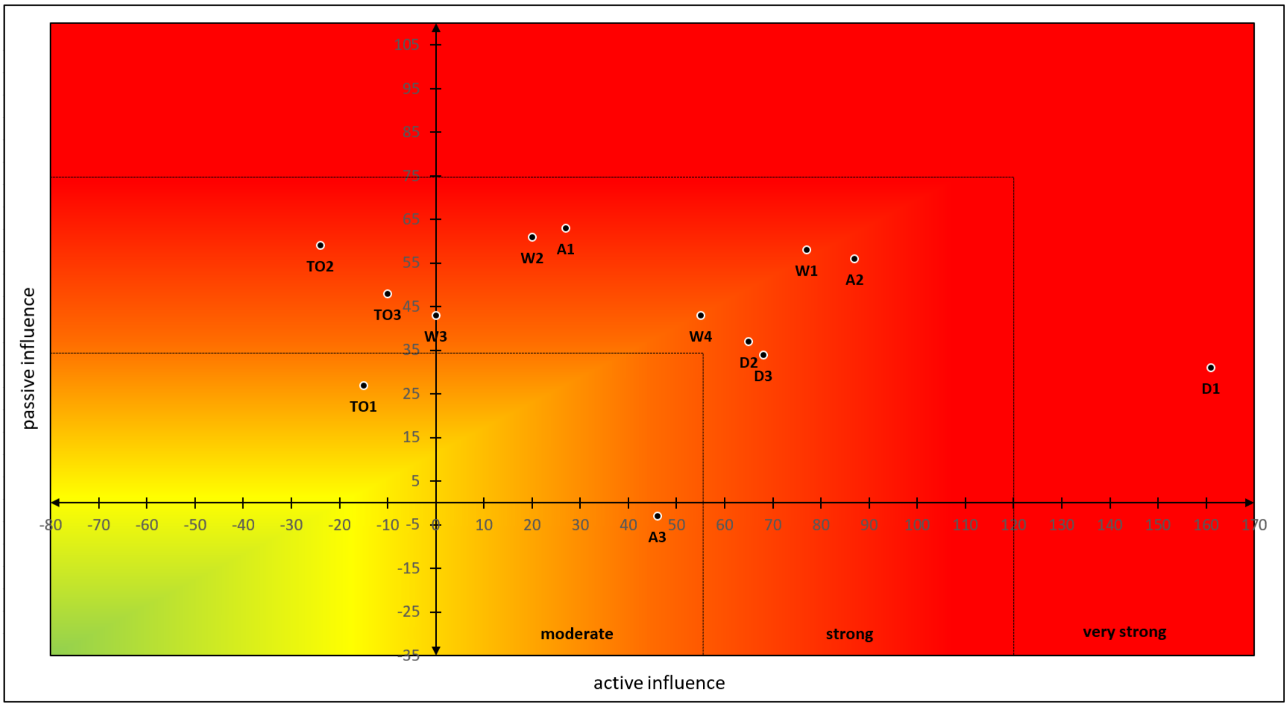

Figure 19c. Differentiation of the Re-Identification risk according to influencing factors.

The application of the quality criteria results in the following assessments.

Solution quality: The score of 1.114 indicates a high risk of possible re-identification. Since several personal references could actually be reconstructed, this assessment is basically plausible, but again classified as too low. However, it should also be noted in this experiment that the distance to the next risk category is very small.

Plausibility: The analysis of the Q-values and the factor differentiation shows once again that the strongest risk driver is the pronounced uniqueness of the datasets (D1). In addition the "skills of the attacker" (A2) and the easily accessible external data material (W1) have a strong influence on the re-identification risk. However, the quality of methods (TO2) has the strongest inhibiting effect on the system.

This result is also consistent with the re-identification risk model in this experiment; the risk drivers identified are comprehensible in terms of their effect. In addition, the Q-value of the entity "attacker's resources" falls into the negative range in this observation, which indicates a risk-reducing effect. This effect is caused by the fact that the resources are consumed by the characteristics of the other entities.

Selectivity: Assuming that the datasets in this experiment had been treated with anonymisation methods after the initial risk assessment, a significant reduction in the overall risk could be determined.

By focusing on the Q-values and the use of methods that

- reduce the number of uniquely configured data records to less than 5% of the total data records (point value 3 → 1),
- increase the number of similar data records due to the first measure (point value 3 → 0),
- limit the number of vulnerable quasi-identifiers to one instance (such as the diagnostic codes; point value 3 → 1),
- meet the criterion of t-closeness (quality of methods; score 1 → 3),
- reduce data inference due to the effects already described (score 3 → 1),

a risk score of 546 points is reached, if the characteristics of the other influencing factors remain unchanged. This shows a clear difference in the risk score before treatment of the datasets and after application of the corresponding measures.

Effort: The assessment of the effort required to apply the RIMEDA procedure can be carried out analogously to the first experiment.

As before, the investigated datasets should be available in a machine-readable format for viewing the entities of the data perspective. Thus, with the support of suitable software, it is also possible to obtain a statement about the characteristics of these features quickly and (except for possible license costs) without financial expense. However, methodological knowledge is essential, i.e. an appropriately trained person has to carry out the assessment.

Since the study in question focused on deaths associated with Covid-19, the use of obituaries for re-identification purposes was obvious and the associated effort is estimated to be low. The characteristics of other sources can only be estimated due to the wide range of possible data sources.

Furthermore, as in the first experiment, the potential for data inference eludes concrete methodological recording. The existence of metadata in the datasets, on the other hand, should be easy to determine. Overall, the resources required to consider the knowledge perspective are estimated to be low, but domain knowledge is necessary to assess data inference.

The entities of the attacker perspective can also only be estimated, for which virtually no effort is required.

The entities of the technical/organisational perspective can be assessed in part through research, but in other cases only indirectly. As the Portuguese Ministry of Health is an authority at the highest administrative level, data protection requirements or similar can be derived from EU regulations. Methods for implementing the organisation's data security are not made publicly available for obvious reasons; however, due to the size and importance of the authority, it can also be assumed that this topic is given a certain weight. In this respect, in addition to domain knowledge in relation to data protection law, time is primarily required to obtain the necessary information.

In summary, the aim of this part was to obtain a statement on the quality of the presented heuristic RIMEDA procedure for stratifying the re-identification risk of medical data.

First of all, quality criteria were presented to be used for the assessment:

- *Solution quality*: How well is the re-identification risk estimated by the heuristic?
- *Plausibility*: Are the identified characteristics of the risk perspectives logical and
- comprehensible in terms of content?
- *Selectivity*: Can the heuristic determine a clear difference in the assumed risk characteristics in different situations?
- *Effort*: What resources are required to apply the heuristic?

To assess the general quality of the heuristic, two experiments were conducted on the basis of two successful, i.e. verified, re-identification attacks [76,78]. The attacked data was retrospectively examined for its risk potential using the information from the publications. The method was then assessed on the basis of the quality criteria presented.

With regard to the solution quality, it could be shown that the heuristic can basically provide an indication of a high risk of an undesired reconstruction of a personal reference.

In view of the plausibility of the method, it became clear that the differentiation of the risk into its components is comprehensible. It also provides valuable information on how to respond to the identified threat situation.
A comparison of the risk level before and after the application of data protection measures shows a clear difference in terms of risk stratification. The discriminatory power is therefore rated as good. The effort involved in applying the heuristic depends on the perspective under consideration. In any case, support from suitable software, methodological knowledge, and medical knowledge, and medical domain knowledge are required.

Meaning of the results of the application tests

Hypothetical risk scenarios were constructed in order to propose a categorisation of the re-identification risk. These represent the maximum and minimum risk of undesired de-anonymisation that can be depicted using this heuristic in the form of a risk score. The range between the maximum and minimum values was then divided into sections of equal size to represent the risk categories.

In the experiments to test the quality of the heuristic, it was noticeable that this classification proposal obviously categorised the re-identification risk as lower than it probably actually was. As already mentioned, the main reason for this underestimation is probably that not all entities could be conclusively determined in the studies used, as some of the relevant information was missing. In addition, there is of course the possibility that the proposed categories do not adequately assess the risk of re-identification and that further evaluations of the heuristics are necessary, which can then be used to improve the categorisation.

In this context, however, it should be pointed out that the suspected misjudgement was only minor, the publications analysed and the data described therein were classified as very risky overall, the distance to the next (and then highest) risk category was small in each case.

References

1. European Union. EUR-Lex. 2016. https://eur-lex.europa.eu/eli/reg/2016/679/oj/eng Accessed 7 Oct 2025

2. El Emam K, Arbuckle L. Anonymizing health data: case studies and methods to get you started. First Edition. Beijing: O'Reilly; 2014.

3. Smith C. Preventing Unintended Disclosure of Personally Identifiable Data Following Anonymisation. In: Informatics for Health: Connected Citizen-Led Wellness and Population Health. Amsterdam, Berlin, Washington, DC: IOS Press; 2017. p. 331-6.

4. Dalenius T. Finding a Needle In a Haystack or Identifying Anonymous Census Records. J Off Stat. Stockholm: Statistics Sweden (SCB); 1986;2:329.

5. Rocher L, Hendrickx JM, de Montjoye Y-A. Estimating the success of re-identifications in incomplete datasets using generative models. Nat Commun. 2019;10:3069.

6. U.S. Department of Health and Human Services. Guidance Regarding Methods for De-identification of Protected Health Information in Accordance with the Health Insurance Portability and Accountability Act (HIPAA) Privacy Rule. Health Information Privacy. 2025. https://www.hhs.gov/hipaa/for-professionals/special-topics/de-identification/index.html#protected. Accessed 7 Oct 2025

7. Wälder K, Wälder O. Der Risikobegriff. In: Wälder K, Wälder O, editors. Methods for risk modeling and risk management [Methoden zur Risikomodellierung und des Risikomanagements]. Wiesbaden: Springer Fachmedien Wiesbaden; 2017. p. 1-3.

8. Helbing K, Ganslandt T, Drepper J, Pommerening K. Guide to data protection in medical research projects: Generic solutions from TMF 2.0 [Leitfaden zum Datenschutz in medizinischen Forschungsprojekten: Generische Lösungen der TMF 2.0]. MWV Medizinisch Wissenschaftliche Verlagsgesellschaft mbH & Co. KG; 2017.

9. Citron DK, Solove DJ. Privacy harms. BUL Rev. 2022;102:793.

10. Lambert D. Measures of Disclosure Risks and Harm. J Off Stat. 1993;9:313-31.

11. Nergiz ME, Atzori M, Clifton C. Hiding the presence of individuals from shared databases. In: Proceedings of the 2007 ACM SIGMOD International Conference on Management of Data - SIGMOD '07. Beijing, China: ACM Press; 2007. p. 665.

12. Duncan GT, Lambert D. Disclosure-Limited Data Dissemination. J Am Stat Assoc. 1986;81:10-8.

13. Li J, Liu J, Baig M, Wong RC-W. Information based data anonymization for classification utility. Data Knowl Eng. 2011;70:1030-45.

14. Alder S. 2024 Healthcare Data Breach Report. HIPAA J. 2024. https://www.hipaajournal.com/2024-healthcare-data-breach-report/. Accessed 16 July 2025

15. Open Definition 2.1 - Open Definition - Defining Open in Open Data, Open Content and Open Knowledge. https://opendefinition.org/od/2.1/en/. Accessed 08 Oct 2025

16. Garfinkel SL. De-identification of personal information. National Institute of Standards and Technology; 2015. Report No.: NIST IR 8053. doi: 10.6028/NIST.IR.8053

17. Schneble CO, Elger BS, Shaw D. The Cambridge Analytica affair and Internet-mediated research. EMBO Rep. 2018;19.

18. Willenborg L, de Waal T. Statistical Disclosure Control in Practice. New York, NY: Springer New York; 1996.

19. De Bruin J. Python Record Linkage Toolkit: A toolkit for record linkage and duplicate detection in Python. Zenodo; 2019. doi: 10.5281/zenodo.3559043

20. Dusetzina SB, Tyree S, Meyer A-M, Meyer A, Green L, Carpenter WR. Linking Data for Health Services Research: A Framework and Instructional Guide. AHRQ Publication No. 14-EHC033-EF. Rockville, MD: Agency for Healthcare Research and Quality; 2014.

21. Hejblum BP, Weber GM, Liao KP, Palmer NP, Churchill S, Shadick NA, et al. Probabilistic record linkage of de-identified research datasets with discrepancies using diagnosis codes. Sci Data. 2019;6:180298.

22. Fung BCM, Wang K, Chen R, Yu PS. Privacy-preserving data publishing: A survey of recent developments. ACM Comput Surv. 2010;42:1-53.

23. Chen B-C, Kifer D, LeFevre K, Machanavajjhala A. Privacy-Preserving Data Publishing. Found Trends® Databases. 2009;2:1-167.

24. Denning DE, Denning PJ. The tracker: a threat to statistical database security. Schwartz MD, editor. ACM Trans Database Syst. 1979;4:76-96.

25. Summa L, Mansmann U, Buchner B, Schnebbe M. Bit Data in Medicine [Big Data in der Medizin]. Freiburg; München: Karl Alber; 2020.

26. Bayardo RJ, Agrawal R. Data Privacy through Optimal k-Anonymization. 21st Int Conf Data Eng ICDE05. Tokyo, Japan: IEEE; 2005. p. 217-28.

27. Wang K, Fung BCM, Yu PS. Handicapping attacker's confidence: an alternative to k-anonymization. Knowl Inf Syst. 2007;11:345-68.

28. Meyerson A, Williams R. On the complexity of optimal K-anonymity. Proceedings of the twenty-third ACM SIGMOD-SIGACT-SIGART Symposium on Principles of Database Systems - PODS '04. Paris, France: ACM Press; 2004. p. 223.

29. Oganian A, Iacob I, Lesaja G. Multivariate Top-Coding for Statistical Disclosure Limitation. In: Domingo-Ferrer J, Muralidhar K, editors. Privacy in Statistical Databases. Cham: Springer International Publishing; 2020. p. 136-48.

30. El Emam K, Dankar FK, Neisa A, Jonker E. Evaluating the risk of patient re-identification from adverse drug event reports. BMC Med Inform Decis Mak. 2013;13:114.

31. El Emam K. Guide to the De-Identification of Personal Health Information. Auerbach Publications; 2013. doi: 10.1201/b14764

32. Reiss SP. Practical data-swapping: the first steps. ACM Trans Database Syst 1984;9:20-37.

33. Wang X, Yu H. How to Break MD5 and Other Hash Functions. In: Cramer R, editor. Advances in Cryptology – EUROCRYPT 2005. Berlin, Heidelberg: Springer Berlin Heidelberg; 2005. p. 19-35.

34. Brand R. Microdata Protection through Noise Addition. In: Domingo-Ferrer J, editor. Inference Control in Statistical Databases. Berlin, Heidelberg: Springer Berlin Heidelberg; 2002. p. 97-116.

35. Kargupta H, Datta S, Wang Q, Krishnamoorthy Sivakumar. On the privacy preserving properties of random data perturbation techniques. Third IEEE International Conference Data Minining. Melbourne, FL, USA: IEEE Comput Soc; 2003. p. 99-106.

36. Sweeney L. Achieving k-Anonymity Privacy Protection using Generalization and Suppression. Int J Uncertain Fuzziness Knowl-Based Syst. 2002;10:571-88.

37. LeFevre K, DeWitt DJ, Ramakrishnan R. Incognito: efficient full-domain K-anonymity. Proc 2005 ACM SIGMOD International Conference on Management of Data - SIGMOD ‘05. Baltimore, Maryland: ACM Press; 2005. p. 49.

38. Wong RC-W, Li J, Fu AW-C, Wang K. (α, k)-anonymity: an enhanced k-anonymity model for privacy preserving data publishing. Proc 12th ACM SIGKDD International Conference on Knowledge Discovery and Data Mining. Philadelphia PA USA: ACM; 2006. p. 754-9.

39. LeFevre K, DeWitt DJ, Ramakrishnan R. Mondrian Multidimensional K-Anonymity. 22nd International Conference on Data Engineering ICDE ‘06. Atlanta, GA, USA: IEEE; 2006.

40. Xiao X, Tao Y. Anatomy: Simple and Effective Privacy Preservation. In: Proceedings of the 32nd International Conference on Very Large Data Bases. Seoul, Korea; 2006. p. 139-50.

41. Li T, Li N, Zhang J, Molloy I. Slicing: A New Approach for Privacy Preserving Data Publishing. IEEE Trans Knowl Data Eng. 2012;24:561-74.

42. Grunert H-F, Heuer A. Slicing in assistance systems - How valuable analysis results can be obtained despite data anonymization [Slicing in Assistenzsystemen - Wie trotz Anonymisierung von Daten wertvolle Analyseergebnisse gewonnen werden können]. Comput Science. Magdeburg, Germany; 2015. p. 24-9.

43. Goncalves A, Ray P, Soper B, Stevens J, Coyle L, Sales AP. Generation and evaluation of synthetic patient data. BMC Med Res Methodol. 2020;20:108.

44. ARX - Data Anonymization Tool | A comprehensive software for privacy-preserving microdata publishing. https://arx.deidentifier.org/. Accessed 09 Oct 2025

45. Samarati P, Sweeney L. Protecting Privacy when Disclosing Information: k-Anonymity and Its Enforcement through Generalization and Suppression. Computer Science Laboratory, SRI International; 1998.

46. Institute of Medicine. Sharing Clinical Trial Data: Maximizing Benefits, Minimizing Risk. Washington, D.C.: National Academies Press; 2015; 18998.

47. Machanavajjhala A, Gehrke J, Kifer D, Venkitasubramaniam M. L-diversity: privacy beyond k-anonymity. 22nd International Conference on Data Engineering ICDE ‘06. Atlanta, GA, USA: IEEE; 2006. p. 24–24.

48. Li N, Li T, Venkatasubramanian S, Labs T. *t*-Closeness: Privacy Beyond *k*-Anonymity and -Diversity. IEEE 2007;106-115.

49. Rubner Y, Tomasi C, Guibas LJ. The Earth Mover’s Distance as a Metric for Image Retrieval. Int J Comp Vision. 2000;40:99-121.

50. Dwork C. Differential privacy. In: Bugliesi M, Preneel B, Sassone V, Wegener I, editors. Automata, Languages and Programming. ICALP 2006. Lecture Notes in Computer Science, vol 4052. Springer, Berlin, Heidelberg.

51. Dwork C, Roth A. The Algorithmic Foundations of Differential Privacy. Found Trends® Theor Comput Sci. 2013;9:211-407.

52. Kohlmayer FM. Data protection and biomedical research: concepts and solutions for anonymity [Datenschutz und biomedizinische Forschung: Konzepte und Lösungen für Anonymität]. Technical University of Munich, TUM; 2015. https://mediatum.ub.tum.de/doc/1280009/884611.pdf. Accessed 08.10.2025

53. Iyengar VS. Transforming data to satisfy privacy constraints. In: Proceedings of the eighth ACM SIGKDD International Conference on Knowledge Discovery and Data Mining. New York, NY, USA: Association for Computing Machinery; 2002. p. 279-88.

54. de Waal T, Willenborg L. Informations loss through global recoding an local suppression. Neth Off Stat. 1999;14:17-20.

55. Ester M, Sander J. Clustering. In: Ester M, Sander J, editors. Knowledge Discovery in Databases: Techniques and Applications [Knowledge Discovery in Databases: Techniken und Anwendungen]. Berlin, Heidelberg: Springer Berlin Heidelberg; 2000. p. 45-105.

56. Howe HL, Lake AJ, Shen T. Method to Assess Identifiability in Electronic Data Files. Am J Epidemiol. 2006;165:597-601.

57. Lee YJ, Lee KH. What are the optimum quasi-identifiers to re-identify medical records? In: 2018 20th International Conference on Advanced Communication Technology (ICACT). Chuncheon-si Gangwon-do, Korea (South): IEEE; 2018. p. 1025-33.

58. Bönisch C, Kesztyüs D, Kesztyüs T. Harvesting metadata in clinical care: a crosswalk between FHIR, OMOP, CDISC and openEHR metadata. Sci Data. 2022;9:659.

59. Mills JL, Harclerode K. Privacy, Mass Intrusion and the Modern Data Breach. Fla Law Rev. 2017;69.

60. Riley J. Understanding metadata: what is metadata, and what is it for? Baltimore, MD: NISO Press; 2017.

61. Mayernik MS, Acker A. Tracing the traces: The critical role of metadata within networked communications. J Assoc Inf Sci Technol. 2018;69:177-80.

62. Hauswaldt J, Demmer I, Heinemann S, Himmel W, Hummers E, Pung J, et al. The risk of re-identification in the evaluation of routine medical data – Critical assessment and possible solutions [Das Risiko von Re-Identifizierung bei der Auswertung medizinischer Routinedaten – Kritische Bewertung und Lösungsansätze]. Z Evid Fortbild Qual Gesundhwes. 2019;149:22-31.

63. Dankar FK, El Emam K, Neisa A, Roffey T. Estimating the re-identification risk of clinical data sets. BMC Med Inform Decis Mak. 2012;12:66.

64. Loukides G, Denny JC, Malin B. The disclosure of diagnosis codes can breach research participants' privacy. J Am Med Inform Assoc. 2010;17:322-7.

65. Janik M, Weber K, Schütz AE, Fertig T. Automated measurement of information security behavior [Informationssicheres Verhalten automatisiert messen]. In: D-A-CH Security 2018: Bestandsaufnahme - Konzepte - Anwendungen - Perspekt. syssec; 2018. p. 1-12.

66. Helisch M, Pokoyski D, editors. Security Awareness. Wiesbaden: Vieweg+Teubner; 2009.

67. Jercich K. The biggest healthcare data breaches of 2021. Healthc. IT News. 2021. https://www.healthcareitnews.com/news/biggest-healthcare-data-breaches-2021. Accessed 08 Oct 2025

68. Tokmetzis D. How your innocent smartphone passes on almost your entire life to the secret service. Bits Freedom. 2014. https://www.bitsoffreedom.nl/2014/07/30/how-your-innocent-smartphone-passes-on-almost-your-entire-life-to-the-secret-service/. Accessed 28 Nov 2024

69. Bönisch C, Schmidt C, Kesztyüs D, Kestler HA, Kesztyüs T. Proposal for Using AI to Assess Clinical Data Integrity and Generate Metadata: Algorithm Development and Validation. JMIR Med Inform. 2025;13:e60204–e60204.

70. Langarizadeh M, Orooji A, Sheikhtaheri A. Effectiveness of Anonymization Methods in Preserving Patients' Privacy: A Systematic Literature Review. Stud Health Technol Inform. 2018;248:80-7.

71. Lauf F, Scheider S, Friese J, Kilz S, Radic, Burmann A. Exploring Design Characteristics of Data Trustees in Healthcare - Taxonomy and Archetypes. In: ECIS 2023 Research Papers. Kristiansand, Norway. p. 323.

72. De Capitani di Vimercati S, Foresti S, Livraga G, Samarati P. k-Anonymity: From Theory to Applications. Trans Data Priv. 2023;16:25-49.

73. Dankar FK, El Emam K. Practicing Differential Privacy in Health Care: A Review. Trans Data Priv. 2013;6:35-67.

74. Faller H. Methodological principles [Methodische Grundlagen]. In: Medizinische Psychologie und Soziologie. Berlin, Heidelberg: Springer Berlin Heidelberg; 2016. p. 51-97.

75. Zimmermann H-J. Heuristic methods [Heuristische Verfahren]. In: Operations Research. Wiesbaden: Vieweg+Teubner Verlag; 2008. p. 271–306.

76. Sweeney L. k-ANONYMITY: A MODEL FOR PROTECTING PRIVACY. Int J Uncertain Fuzziness Knowl-Based Syst. 2002;10:557-70.

77. Sweeney L. Simple Demographics Often Identify People Uniquely. Pittsburgh: Carnegie Mellon University; 2000.

78. Carvalho T, Faria P, Antunes L, Moniz N. Fundamental privacy rights in a pandemic state. PLOS ONE. 2021;16:e0252169.
